# Brightness of fluorescent organic nanomaterials

**DOI:** 10.1039/d2cs00464j

**Published:** 2023-06-20

**Authors:** Anila Hoskere Ashoka, Ilya O. Aparin, Andreas Reisch, Andrey S. Klymchenko

**Affiliations:** a Laboratoire de Bioimagerie et Pathologies, UMR 7021 CNRS, Université de Strasbourg 74 route du Rhin 67401 Illkirch France andrey.klymchenko@unistra.fr

## Abstract

Brightness is a fundamental property of fluorescent nanomaterials reflecting their capacity to absorb and emit light. In sensing materials, brightness is crucial for high-sensitivity (bio)molecular detection, while in optical bioimaging it ensures high spatial and temporal resolution. Fluorescent organic nanoparticles (NPs) are particularly attractive because of their superior brightness compared to organic dyes. With the ever-growing diversity of organic nanomaterials, it is important to establish universal principles for measuring and estimating their brightness. This tutorial review provides definitions of brightness and describes the major approaches to its analysis based on ensemble and single-particle techniques. We present the current chemical approaches to fight Aggregation-Caused Quenching (ACQ) of fluorophores, which is a major challenge in the design of bright organic nanomaterials. The main classes of fluorescent organic NPs are described, including conjugated polymer NPs, aggregation-induced emission NPs, and NPs based on neutral and ionic dyes. Their brightness and other properties are systematically compared. Some brightest examples of bulk solid-state emissive organic materials are also mentioned. Finally, we analyse the importance of brightness and other particle properties in biological applications, such as bioimaging and biosensing. This tutorial will provide guidelines for chemists on the design of fluorescent organic NPs with improved performance and help them to estimate and compare the brightness of new nanomaterials with literature reports. Moreover, it will help biologists to select appropriate materials for sensing and imaging applications.

## Abstract 2

We introduce and analyse brightness of fluorescent organic nanomaterials as an essential characteristic for biosensing and bioimaging applications.

## Graphical abstract

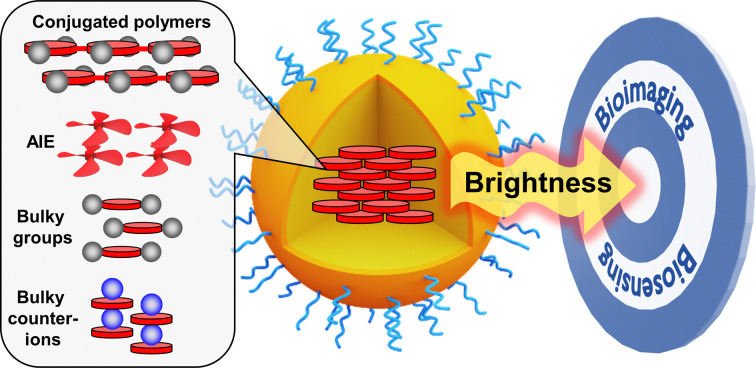

**Key learning points**

(1) Definition of brightness and methods for its estimation for fluorescent nanomaterials at the ensemble and single-particle level.

(2) Approaches to prevent aggregation-caused quenching in order to design bright organic nanomaterials.

(3) Key classes of bright organic nanoparticles with systematic comparison of their brightness.

(4) Insights on the choice of fluorescent NPs for biosensing and bioimaging applications.

(5) Current challenges and perspectives in bright organic nanomaterials.

## 1. Introduction

**Fig. 1:**
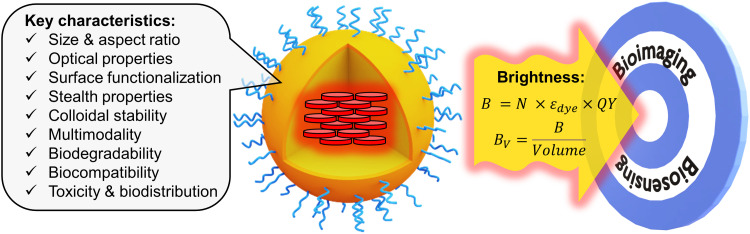
Fluorescent organic NPs, their key characteristics with focus on brightness.

Even though the fluorescence imaging field has been dominated since quite some time by fluorescent dyes^1^ and fluorescent proteins,^2^ they present a fundamental limitation in terms of fluorescence brightness. In chemistry, the fluorescence brightness is defined as a product of molar extinction coefficient and fluorescence quantum yield, which for molecular emitters is physically limited to ∼300 000 M^−1^ cm^−1^.^1,3^ Brightness is a key property that defines the number of photons that can be collected for a given time period from the fluorescent probe. It determines the detection sensitivity in biosensing as well as spatial and temporal resolution in bioimaging.^1^ The limitations of classical fluorescent molecular and biomolecular probes in terms of brightness have stimulated the development of nanoscale materials (nanoparticles, NPs), because their molar extinction coefficient can be 10–1000-fold higher than that of molecular dyes.^4,5^ Among them, particularly attractive in terms of flexibility, rich surface chemistry and biocompatibility are fluorescent organic NPs,^6^ such as conjugated polymer NPs,^7–9^ aggregation-induced emission (AIE) NPs,^10–12^ dye-loaded polymeric NPs,^5,13^*etc.* Fluorescent organic NPs can be defined as fluorescent nanoscale materials composed mainly (if not exclusively) of organic components, in contrast to other types of luminescent NPs, such as quantum dots (QDs),^14^ dye-loaded silica NPs,^15^ metal nanoclusters,^16^ metal–organic framework NPs,^17^ carbon dots,^18^*etc.* There are several excellent reviews focused on the synthesis and applications of fluorescent organic nanomaterials.^5–8,10,11,13,19^ These reviews highlight the importance of critical characteristics of fluorescent organic NPs, such as size, aspect ratio, optical properties, surface functionalization, stealth properties, colloidal stability, multimodality, biodegradability, biocompatibility, toxicity and biodistribution (Fig. 1). In this tutorial review, we will focus on the fluorescence brightness of organic NPs, which has not yet been systematically addressed in the current literature. With the large variety of developed fluorescent organic NPs, the lack of a common comparative approach in terms of brightness makes it difficult to choose the proper tool for sensing and imaging applications. Moreover, brightness determination is a complex issue, which often leads to misinterpretation and incorrect comparison between fluorescent NPs of different nature. This tutorial review will provide a clear methodology for theoretical and experimental estimation of brightness of organic NPs. It will also describe basic concepts for designing bright nanomaterials, in particular, how to prevent the fundamental problem of aggregation-caused quenching (ACQ) of dyes in organic nanomaterials.^5,10^ Then, a systematic comparison will be given for key classes of organic NPs in terms of brightness and structural characteristics, such as size and intrinsic organization of emitters within the nanomaterial. We will also discuss other characteristics of nanomaterials, affecting the performance of fluorescence biosensing and bioimaging: (i) absorption and emission wavelength, which should be shifted to the red in order to achieve deeper tissue penetration and lower photodamage; (ii) photostability should be high in order to collect a maximum of photons; (iii) ON/OFF switching (blinking), which should be suppressed for tracking application or used for super-resolution microscopy; (iv) compatibility with two-photon excitation, which is particularly suitable for tissue imaging. Finally, we will provide insight on how brightness, other optical properties and size of NPs define their biological sensing applications. Overall, we provide a tutorial for chemists, physicists and biologists, to facilitate the design of new NPs, estimate their brightness and compare it with existing NPs, and further choose the right fluorescent tool for a given biological application.

## 2 Brightness of fluorescent organic nanoparticles

### 2.1 Definitions of brightness

On a fundamental level, fluorescence brightness refers to the number of photons emitted by a probe per unit time upon irradiation with a given irradiance (excitation power density), where the irradiance corresponds to the power per surface. For practical reasons, the brightness is expressed in various ways depending on the context and experimental method used.^20,21^ In the chemical sciences, it is expressed based on molar extinction coefficient (*ε*) and quantum yield (QY) of an emitter:

1 Brightness = *ε* × QY

typically in units of M^−1^ cm^−1^.^3^

The quantum yield is expressed as the following:2
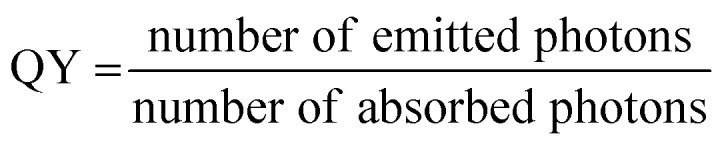
In physical sciences, absorption cross-section *σ* is generally used instead of extinction coefficient, typically in units of length^2^ (*e.g.* cm^2^). In consequence, fluorescence brightness can be calculated as a fluorescence cross-section (or fluorescence excitation cross-section) according to:

3 *σ*_fluo_ = *σ* × QY

which has again dimensions of length^2^. If *ε* is expressed in units of M^−1^ cm^−1^, and *σ* in units of cm^2^, the two can be converted according to:

4 *σ* = 3.82 × 10^−21^ × *ε*

In case of nanomaterials composed of multiple emitters (dyes), their brightness (*B*) depends on the number of emitters per NP, as follows:

5 Brightness (*B*) = *N* × *ε*_dye_ × QY

where *ε*_dye_ is the extinction coefficient of one dye (emitter), *N* is the number of emitters per NP, and QY the quantum yield of the nanomaterial. Here, we assume that the total extinction coefficient of NP is a simple sum of the emitters. As it will be explained below, aggregation of dyes in the solid state may decrease the actual *ε*_dye_, although this deviation is often not dramatic.

Here, the brightness is an ensemble value, which reflects the mean brightness of a large population of NPs, typically also averaged over relatively long measurement times (in the range of tens of seconds to minutes for most spectrofluorometers). On the other hand, brightness can be directly estimated in single-particle measurements^22^ either with respect to a reference emitter (relative brightness) or by precisely estimating the excitation and emission photon flux (also called light power density or irradiance) from single particles (absolute brightness).^23^ In the latter case the single particle brightness can, for example, be defined as:^24^6
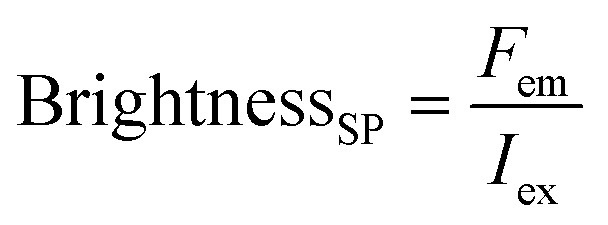
where *F*_em_ is the number of emitted photons per second and *I*_ex_ is the irradiance. When the latter is given in W cm^−2^, as often the case for laser illumination, the single particle brightness can be expressed in7
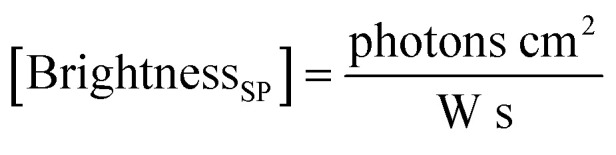
In order to obtain a more easily measurable quantity, Scheblykin and co-workers have defined a single-molecule brightness (*B*) according to:8
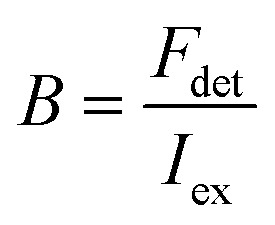
where *F*_det_ is the number of detected photons per second. The *B* value is therefore dependent on the used set-up, and, in particular, on its light detection efficiency. However, results from different set-ups can be compared if a given standard dye or nanoparticle is used.

In the case of organic NPs, the particle size has a direct influence on the brightness. For a given loading of the NPs with dyes, the number of dyes per NP, is proportional to the particle volume, which means to the power of three of the diameter. If there is no major influence of the size on the QY, this then also means that the brightness is proportional to volume. This behaviour is expected for all types of NPs, where the dye is encapsulated inside the NPs, for example in dye-loaded polymeric NPs, AIE NPs or conjugated polymer NPs. One should note that variation of NP size in this case does not have a direct effect on the absorption and fluorescence spectra of NPs. This is different from some inorganic NPs like QDs, nanodiamonds, carbon dots, *etc.*^21^

In order to compare the inherent brightness of fluorescent nanomaterials of different sizes, it is therefore interesting to define a brightness per volume (*B*_V_):9
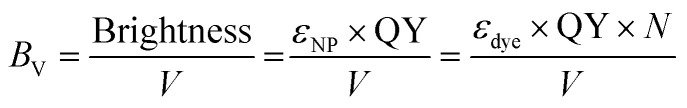
where *N* is the number of fluorescent dyes encapsulated inside the NPs and *V* is the volume of the particle (nm^3^). Here, *B*_V_ will be given in units of M^−1^ cm^−1^ nm^−3^.

In case of dye-based NPs, *N* can be expressed as follows:10
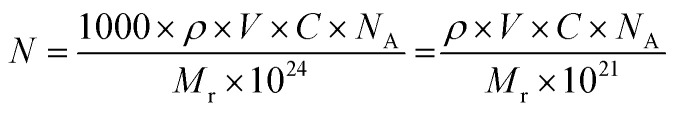
where *ρ* is the density of the NP material (g mL^−1^), *V* is the volume of NP (nm^3^), *C* is the mass fraction of the dye (for pure dye it is 1), *N*_A_ is the Avogadro number and *M*r is the molecular weight of the dye. Then combining the eqn (9) and (10), we can obtain the expression of brightness per volume independently of the volume of NPs:11
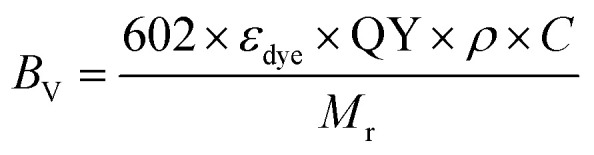
This equation provides a simple estimation of *B*_V_, given that it does not require the knowledge of exact NP size. However, the density (*ρ*) of NP material is not always easy to assess, although for most organic materials it can be assumed to be of the order of 1 g mL^−1^. It should be noted that *B*_V_ is useful to compare efficiency of materials of different type and size, but only the total brightness *B* defines the performance of NPs in practical applications. Indeed, the *B*_V_ value of organic materials is the highest for dyes in molecular form, because of their very small volume, whereas their total brightness is far lower than that of organic NPs (see below).

Another point to be considered is the appearance of inner filter effects that can occur at very high local dye concentrations inside NPs even at the level of a single particle. According to the Beer–Lambert law the absorbance is expressed as follows:

12 *A* = *ε*_dye_ × *l* × *c*

where we can assume that *l*, the optical path length, in the first approximation, to be equal to the particle diameter (*d*) and *c* to be the molar concentration of the dye inside the particle. By considering, for example, a dye loading of 50 wt%, a dye molar mass of 500 g mol^−1^, a dye molar extinction coefficient of 10^5^ M^−1^ cm^−1^ (corresponding for example to rhodamines) and a global particle density of 1 g mL^−1^, a 100 nm particle diameter would lead to an absorbance of 1. This means that in this case, 90% of the incident light is absorbed by a single NP, corresponding to a very strong inner filter effect within the particle. In consequence, a further increase in the dye loading would not lead to a significant brightness improvement. This consideration implies an upper limit of brightness for fluorescent NPs of a given size, which for 100 nm NPs is of the order of 3 × 10^10^ M^−1^ cm^−1^ according to eqn (5). Moreover, due to high inner-filter effects at high dye loadings in relatively large NPs, the particle brightness would not increase as a power three of the diameter, so that for larger NPs the per-volume brightness would decrease with the particle size.

Furthermore, the influence of the particle size on the QY should be considered. On the one hand, larger size decreases surface to volume ratio, which could decrease the fraction of species quenched by interaction with aqueous medium. On the other hand, larger NPs may favour quenching processes linked to energy transfer effects occurring inside the NPs. Studies in our group on dye-loaded polymeric NPs showed no clear influence of the particle size on the QY.^25^ However, systems that allow to study precisely this effect without varying other parameters are rare.

It should be noted that most of the definitions of brightness given above could be applied to other types of nanomaterials, such as quantum dots, carbon dots, or metal–organic framework NPs. However, these materials cannot be always regarded to contain several individual emitters, and, therefore, their molar absorption coefficient is estimated for the whole particle.

Finally, the particle size is important, because most of biological applications require bright NPs of the smallest possible size (see below). Therefore, both particle brightness and *B*_V_ will be further used in the review to analyse brightness of most representative examples of NPs reported in the literature.

### 2.2. Methods for measuring brightness

The methods to determine brightness experimentally can be distinguished depending on whether they are based on ensemble or single-particle measurements. While the ensemble methods are relatively simple to implement, they rely on several assumptions. Single-particle methods, on the other hand, are more demanding to realize, but they give access to the actual particle brightness and its distribution, in conditions similar to those in which these probes are used in optical microscopy experiments.

#### 2.2.1. Particle brightness by ensemble measurements

Measuring the ensemble brightness of fluorescent nanomaterials requires, according to eqn (1), determining both the extinction coefficient and the quantum yield of the nanomaterial. In case of dye-based NPs, the easiest access to determine the NP extinction coefficient uses the additivity of the absorbance according to eqn (8):

13 *ε*_NP_ = *N* × *ε*_dye_

A typical assumption made in this case is that the extinction coefficient of the encapsulated dyes is close to those of the free dyes in solution, which can be found in literature or easily measured. However, in case of strong hypochromic effects produced by dye aggregation (see below), actual extinction coefficients of dyes within NPs should be measured.

Determining the number *N* of dyes (or emitters) per NP is less straightforward.^26^ In principle, *N* can be determined if the concentration of the dyes in the NPs and the particle size are known:

14 *N* = [dye] × *V*_NP_

In case of composite NPs loaded with dyes, for instance dye-loaded polymeric NPs, the concentration inside the particle can often be estimated based on the used ratios of dye and polymer and the encapsulation efficiency. The size used to calculate the per particle volume should be based on measurements yielding the core particle size rather than the hydrodynamic diameter. For these reasons, size measurements using transmission electron microscopy (TEM) or atomic force microscopy (AFM) are preferred to dynamic light scattering (DLS) measurements.^27^ Direct measurements of the per particle extinction coefficient based on the Beer–Lambert law (*A* = *ε* × l × *c*) can also be used, but require to know precisely the molar concentration of NPs, which can be estimated based on the mass concentration and the particle size. An elegant way to measure molar concentration of NPs is to use fluorescence correlation spectroscopy (FCS), which is a solution-based single-molecule detection method that measures the number, brightness and diffusion coefficient of emissive species in a focal volume.^28,29^ Moreover, for particle suspensions, absorption should be distinguished from scattering, which depends on the particle size and the wavelength used for the excitation. In order, to properly take into account scattering effects, an ideal solution is to determine the optical density of NP suspensions made from the same type of material and having the same size, but being devoid of the chromophore. In cases where this is not possible, a correction for the scattering can be achieved by applying a suitable baseline, for example using a curve ∼*λ*^−4^.

A variety of approaches available to determine the quantum yields of fluorescent NPs have been reviewed in detail previously.^30^ Relative methods remain the most used and, *a priori*, the easiest. In this case, the fluorescence intensity integrated over the whole emission range I_NP_ and absorbance A_NP_ at the excitation wavelength of the NPs are compared to those of a reference dye, *I*_R_ and *A*_R_, respectively, measured under the same spectrofluorometer settings.15
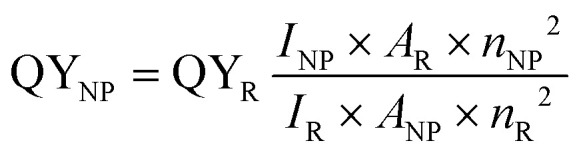
where *n* is the refractive index of the media used for the measurement. The QYs of reference compounds for a large variety of wavelengths are now available.^31,32^ In order to obtain reliable results, the absorption of the samples should be kept below 0.1 (or even 0.05) to avoid inner filter effects. A major contribution to uncertainties of the obtained QY values are scattering and reabsorption, occurring especially in the case of larger particles, though these are not significant for NPs below 100 nm.^30,31^

An alternative is the determination of absolute QYs, which can, for example, be obtained using an integrating sphere.^30^ Integrating spheres collect all photons absorbed, scattered and emitted by the sample and can nowadays be implemented in standard spectrofluorometers. However, a precise calibration of the set-up is required to achieve a high precision.

A general limitation of these approaches is that they typically do not allow obtaining any information on the distribution of particle brightness over the individual nanoparticles.

#### 2.2.2. Brightness by single-particle methods

**Fig. 2:**
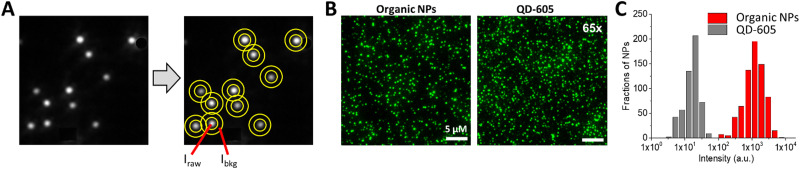
Single-particle brightness analysis. (A) Simplified analysis workflow that includes: detection of local maxima, defining and measuring the intensity at regions of interest around maxima (raw signal: *I*_raw_) and around central regions (background: *I*_bkg_). The signal is then obtained by subtraction of the background from the raw signal. (B,C) Example of single-particle imaging by wide-field microscopy (B) and intensity analysis (C) for 20 nm DNA-modified dye-loaded polymeric NPs (organic NPs) *vs.* reference particles QD-605. In case of QD-605, the source irradiance was increased 65-fold to achieve comparable intensity with organic NPs. Other experimental settings were identical. Excitation wavelength was 550 nm. Panels B and C reproduced with permission from ref. 36. Copyright American Chemical Society.

Single-particle measurements, which are usually done by fluorescence microscopy, address properties of individual particles one by one, in contrast to ensemble measurements done for an entire population of NPs. They provide access to the distribution of the brightness, which depends, on the one hand, on the distribution of particle sizes, but can also be influenced by varying levels of encapsulation and differences in the QYs. For this purpose, the NPs are typically immobilized either on a surface (*e.g.* glass) or in a gel (*e.g.* alginate or polyvinyl alcohol). Negatively charged NPs can be readily immobilized on glass surfaces pre-treated with cationic polymers, such as poly(ethylene imine);^24,33^ whereas for biotinylated NPs (including commercially available QDs),^34,35^ the glass surface is pre-treated with BSA–biotin and then neutravidin, while for DNA-functionalized NPs, biotinylated complementary strands can be deposited on the glass surface pre-treated with BSA–biotin and then neutravidin.^35^ Care has to be taken to achieve sufficient separation of the NPs and to avoid formation of aggregates during adsorption to ensure that actual single particles are imaged. In our studies, we typically worked on pre-treated glass surfaces with NPs at concentrations in the range of 1 to 10 pM,^24,33^ which corresponds to those used for single-molecule imaging. Ideally, methods such as TEM or AFM should be used together with fluorescence microscopy to confirm good separation of the NPs.^33^ Then, the images of NPs of interest should be recorded under well-defined excitation (laser wavelength and excitation irradiance) and collection conditions (wavelength range/filter sets, exposure time, camera settings). Primarily, a semi-quantitative analysis of the brightness of NPs can be made by comparison with reference NPs at equal instrumental settings. For example, comparison with commercial quantum dots (QDs) of similar emission wavelengths can be made (Fig. 2).^24,36^ However, if the brightness of NPs of interest is more than an order of magnitude higher than that of the reference NPs (*e.g.* QDs), imaging settings should be adjusted, for instance by increasing the excitation irradiance for the latter by a given factor (*e.g.* 10 or 20×).^24,35^ One should note that extinction coefficient of QDs increases strongly for shorter wavelengths, therefore the excitation wavelength should be explicitly mentioned.

Next, the image analysis is performed, where the signal of the individual particles (or luminescent spots) is first measured and the background is subtracted (Fig. 2). Simple ImageJ algorithms allow automatic localisation of the brightest spots in an image. Then, a circular region of interest (ROI) with fixed area is defined around the determined localizations and the mean (or total) intensity in the ROI is measured.^24^ Ideally, the local background signal can be subtracted by measuring the intensity in a band around the ROI (Fig. 2). Further, from this data, the mean intensity and the intensity histograms can be generated by analysing hundreds of NPs of interest and reference NPs. An example of such analysis is provided for DNA-modified dye-loaded polymeric NPs in Fig. 2.^36^

Generally, the relative particle brightness depends on the reference and to some extent the instrumental settings. Therefore, attempts have been made to introduce procedures to measure absolute brightness of nanomaterials. In particular, a brightness parameter *B* defined by eqn (8) requires determining the number of detected photons *F*_det_, established based on the microscope and camera parameters.^23,37^ It is possible to go one step further and determine the single-particle brightness as defined by eqn (6),^24^ which is, in principle, independent of the microscope settings and allows comparison of nanomaterials, despite using different setups. However, this approach requires measuring precisely the excitation irradiance, which is done by dividing the irradiation power at the sample position by the illuminated surface area for a given objective. Moreover, it requires converting the recorded fluorescence signal into the emitted photon flux. In this case, the number of detected photons per second *F*_det_ is determined for a given spot according to:16
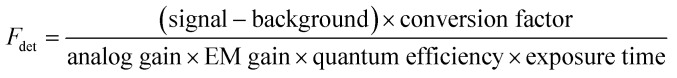
where the conversion factor (electrons/counts) and quantum efficiency (for the given wavelength region) are camera dependent parameters that can be obtained from the manufacturer manual, while the analog and EM gain depend on the experimental settings. The number of emitted photons per second is then obtained according to:17
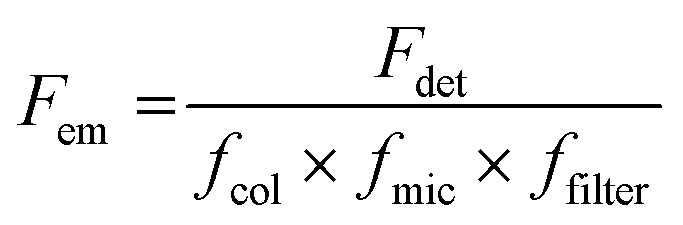
where *f*_col_, *f*_mic_, *f*_filter_ are, respectively, the fraction of photons transmitted due to the collection angle (depends on the numerical aperture of the objective), the microscope set-up (measured independently with direct illumination), and filter settings *vs.* the emission spectrum of NPs. Theoretically, absolute measurements would be the most suitable brightness parameter for comparison of brightness of nanomaterials, obtained in different laboratories. Unfortunately, this approach is difficult to realize because it requires precise determination of all setup parameters, which are not always easily accessible. Therefore, so far, the relative single-particle brightness remains a good compromise for brightness quantification, however, it would require the use the same reference NPs (*e.g.* QDs) by different groups.

A method apart to measure single-particle brightness is based on FCS.^38^ However, it is rarely used, because it requires a dedicated FCS setup, and, moreover, the excitation power used in the focal spot is very high and the particle size should be much smaller than the focal spot.

Generally, the brightness measured at the single-particle level correlates with that measured for a particle ensemble in solution. For example, we showed that single-particle brightness of dye-loaded NPs *vs.* reference NPs, *e.g.* QDs, could be predicted based on the estimations of their extinction coefficient and quantum yield.^24,35,36^ However, the estimations never gave perfect match between the two methods. The primary reason for this is that the excitation power density (irradiance) in the single-particle microscopy technique (∼1 W cm^−2^) is ∼1000-fold higher than that used in the fluorometer (∼1 mW cm^−2^). This high irradiance can produce saturation effects inside NPs as well as photobleaching, which will contribute to the decrease in the actual brightness. The second reason is the difference in the detection optics, which generally requires dichroic mirrors and filters in the case of fluorescence microscope, which should be taken into account when the NPs of interest are compared with the reference NPs. Therefore, for new fluorescent nanomaterials it is always better to estimate both ensemble and single-particle brightness, which would facilitate in the future comparison between different nanomaterials.

## 3. Brightness and aggregation-caused quenching: basic design concepts of fluorescent NPs

**Fig. 3:**
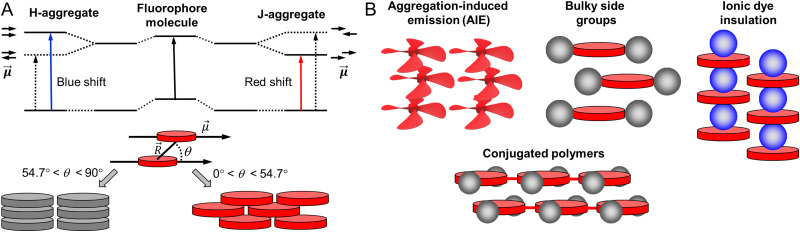
Principles of dye aggregation based on Kasha's exciton theory (A) and the most established methods to prevent ACQ in fluorescent organic nanomaterials (B).

To achieve high brightness in fluorescent nanomaterials, one needs to assemble a large number of dyes, having high fluorescence quantum yield and extinction coefficient, in the small volume of a nanoparticle. However, at high local concentration within nanomaterials, organic dyes, which are usually flat aromatic structures, tend to form non-emissive pi-stacked aggregates with face-to-face assembly. This process, called aggregation-caused quenching (ACQ), is a major challenge in preparation of bright organic nanomaterials. According to the exciton theory, adapted by Kasha to organic fluorophores,^39,40^ dye assemblies could be classified into H- and J-aggregates. These aggregates can be described by the Coulombic intermolecular coupling (*J*_c_), which, in case of parallel dye dipoles, are given by the following equation:^40^18
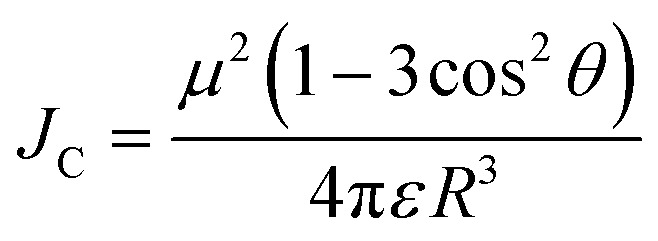
where, *μ* is the dipole moment of a dye, *ε* is the dielectric constant of the medium and *R* is the displacement vector connecting the molecular mass centers of the dyes. In case of J-aggregates, dipoles maintain a head to tail orientation where *θ* is less than the magic angle (0° < *θ* < 54.7° degrees, Fig. 3), leading to negative *J*_C_ values, whereas in case of H-aggregates, dipoles maintain a side-by-side orientation and the angle *θ* is greater than the magic angle (54.7° < *θ* < 90°) leading to positive *J*_C_ values. In the simplest case of a molecular dimer, the Coulomb coupling *J*_C_ leads to the formation of two delocalized excited states split by 2|*J*_C_|: in-phase and out-of-phase states (Fig. 3). The in-phase state, shifted by *J*_C_, is characterized by an enhanced transition dipole moment relative to the monomer, whereas the out-of-phase state, shifted by −*J*_C_, is optically dark because the transition dipoles cancel one another. Therefore, in J-aggregates, the negative *J*_C_ values result in an allowed in-phase transition with lower energy (red shifted) compared to the monomer, whereas in the H-aggregates with *J*_C_ > 0, the allowed in-phase transition is of higher energy (Fig. 3).

Importantly, the emission of J-aggregate is allowed, whereas that of the H-aggregate is forbidden (Fig. 3). H-Aggregation is the most common cause of ACQ in nanomaterials based on dyes. Moreover, H-aggregation leads to hypochromism, which is a reduction of the molar extinction coefficient of dyes within the aggregate, that additionally decreases the brightness of the nanomaterials. Other mechanisms of ACQ should be considered, which include excimer formation, excited state intermolecular charge and electron transfer^20^ within the aggregated dyes as well as inner filter effect within large dye ensembles (see above). J-Aggregates of dyes are attractive for building emissive bulk materials,^41^ however their tendency to form 1D nanostructures makes it difficult to assemble fluorescent NPs and their Stokes shift is too small for conventional bioimaging. Therefore, to prevent ACQ in fluorescent nanomaterials, one should go beyond H- and J-aggregation, which implies control of dye–dye spacing and orientation (Fig. 3).

We can identify the following major approaches to prevent ACQ in organic nanomaterials. First, one should mention the use of conjugated polymers.^42,43^ In this case, the fluorophores are made of conjugated units, which are aligned along the conjugated polymer chain. The spacing between fluorophore units is controlled by the rigid pi-conjugated bonds. Moreover, bulky side groups on these fluorophore units prevent pi-stacking and thus ensure formation of emissive particles. The other approaches to prevent ACQ concern specially designed fluorescent molecules, *i.e.* dyes assembled into nanomaterials without covalent pi-conjugation between them. One of the most popular approaches is the use of aggregation-induced emission (AIE) dyes, proposed by Tang and co-workers in 2001.^44^ In this case, the fluorophore is twisted along its conjugated structure, which generates a propeller-like topology. AIE dyes (AIEgens) are poorly emissive in solution, whereas they light up in the aggregated state because their intramolecular rotation is restricted, while their propeller topology prevents formation of H-aggregates (Fig. 3). The AIE concept gave rise to a great variety of highly emissive (nano)materials for a variety of applications, especially in bioimaging.^10,45^ Alternatively, conventional fluorophores could be redesigned in order to prevent ACQ. The primary approach is to introduce bulky side groups into organic fluorophores. In contrast to AIE, the flat conjugated structure of the dye is maintained (or disturbed to a minor extent), whereas the side groups create a steric hindrance against pi-stacking and H-aggregation.^46–48^ However, this method needs multistep organic synthesis and it cannot be simply transposed to different conventional dyes. Finally, a promising approach to prevent ACQ is based on bulky hydrophobic counterions as insulators of charged organic fluorophores (Fig. 3).^5,33,49^ The large diameter of these counterions (around 1 nm) ensures good spacing between fluorophores and thus prevents their H-aggregation. The approach is particularly suitable for cationic cyanines and rhodamines, which are known for their outstanding brightness and photostability. Importantly, this approach can work in both pure dye salts^50–53^ and in dye-loaded polymeric nanoparticles.^5,33^ Below, we will discuss all these approaches for different classes of organic nanomaterials and analyse their optical properties with focus on the achieved brightness. The structural and spectroscopic data of nanomaterials, discussed in the review, are presented in Table 1.

**Table 1:** Properties of fluorescent organic NPs discussed in this work with some examples of dyes and QDs^a^

Composition	NPs diameter (nm)	Dye content (wt%)	*λ*_abs_ (nm)	*λ*_em_ (nm)	QY (%)	*B* (M^−1^ cm^−1^)	*B*_V_ (M^−1^ m^−1^ nm^−3^)	Singe NP brightness	Ref.
**Molecular organic dyes**									
Rhodamine 6G			530	552	90	1.0 × 10^5^	1.0 × 10^5^		31
Quantum dots									
QD-585				585	60	1.8 × 10^5 ^b			
QD-605				605	52	3.0 × 10^5 ^c			54
QD-705				705	49	1.0 × 10^6 ^c			54
**Conjugated polymer NPs**									
MEH-PPV	15	100	485	590	1	1.2 × 10^6^	651		55
PFBT	15	100	450	545	7	5.1 × 10^5^	2900		55
PFBT	10	100	450	545	30	3 × 10^6^	5700	30 × QD-565	56
PFPV	15	100	445	510	8	1.2 × 10^7^	6520		55
PFBT-DBSOC6 10-COOH	23	50	455	712	15	1.1 × 10^7^	1760	2 × QD-705	57
PF-TC6FQ-BODIPY	26	100	495	723	33	1.7 × 10^7^	1954	3 × QD-705	58 and 59
Pttc-TFQ-BODIPY	28	100	490	724	51	5.1 × 10^7^	4480	7 × QD-705	60
PF-TC6FQ	21	100	493	652	47	2.4 × 10^7^	5070	8 × QD-655	61
PFDBT5–PFBT	15	80	450	650	56	1.6 × 10^7^	9500	15 × QD-655	42
PFGBDP/PFDHTBT–BDP720	31	100	528	721	42	1.8 × 10^8^	11 300	83 × QD-705	62
**Aggregation-induced emission NPs**									
TPETPAFN	33	—	510	670	25	1.1 × 10^7^	560	10 × QD-655	63
BTPEBT	30	33	422	547	63	3.5 × 10^7^	1980		64
BTPEBT-V2	29	40	418	547	62	2.8 × 10^7^	2870		65
BTPEBD	32	33	436	574	90	5.3 × 10^7^	3100		66
PTZ–BT–TPA	100	33	483	656	39	2.4 × 10^9^	4610		67
DTPA–TBZ	50	33	652	929	11	4.0 × 10^7^	614		68
**NPs based on conventional dyes**									
Nile red/PS	100	0.70	570	635	23	7 × 10^7^	134		69
DiD/PLGA	66	0.72	650	667	21	1.3 × 10^7^	84		70
**NPs based on dyes bearing bulky side groups**									
Mes-BODIPY/PS	16	3.5	526	540	77	5.1 × 10^6^	2300		71
BODIPY/PS-PEG	60	1.3	529	544	35	1.4 × 10^8^	470		72
BDP4–PEG(1000)–PMAO	14	18	532	560	60	2.5 × 10^6^	1740	5 × QD-585	73
PDI-Cl/co-polymerized	40	2.4	520	550	50	1.0 × 10^7^	310		74
LR/PLGA	38	5.0	575	605	50	7.5 × 10^6^	261	18 × QD-585	75
**NPs based on charged dyes with bulky counterions**									
R12/F5-TPB	14	100	560	580	32	2.7 × 10^7^	18 700		76
R12/F12-TPB	19	100	560	580	60	6.8 × 10^7^	19 100	45 × QD-585	76
R18/F5-TPB/PLGA	40	5	560	580	20	1.8 × 10^7^	600	6 × QD-605	33
R18/F5-TPB/PMMA-MA	15	5	560	580	60	3.0 × 10^6^	1600	10 × QD-585	77
R18/F5-TPB/PMMA-MA	34	23	560	580	31	8.9 × 10^7^	4330	100 × QD-585	24
R18/F5-TPB/PCL	33	23	560	580	34	8.9 × 10^7^	4730		24
R18/F5-TPB/PMMA-N3	40	23	560	580	46	1.9 × 10^8^	5790	100 × QD-605	35
R18/F5-TPB/PEMA-N3	20	33	560	580	52	3.9 × 10^7^	9480	87 × Q-605	35
BlueCy/TPB/PMMA-MA	40	12	425	475	17.3	2.3 × 10^7^	687	70 × QD-525	78
R6G-C18/F12-TPB/PMMA-N3	44	250 mM	530	570	23	1.1 × 10^8^	2490		79
Cy5/F12TPB/PEMA-N3	16	23	652	682	42	9.7 × 10^6^	4530	22 × QD-705	54
PhSP18/F5-TPB/PMMA-MA	40	29	507	675	40	1.2 × 10^7^	3840	50 × QD-605	80
DiI/TPB/lipid droplets	87	8	553	575	14	2.5 × 10^8^	725		81
Cyanostar	16	—	560	580	30	1.5 × 10^7^	5000^d^	20 × FS	82

a *B* and *B*_V_ are brightness and per-volume brightness of NPs estimated based on eqn (5) and (9) or (11), respectively. For rhodamine 6G we assume the volume of the molecule of 1 nm^3^. Single NP brightness is experimental value obtained from single-particle microscopy measurements. The brightness of QD-685, QD-605 and QD-705 was estimated for the following excitation wavelengths: 488.

b 532.

c 532 (c) nm, respectively.

d Data from ref. 82.

## 4. Classes of fluorescent organic nanoparticles

### 4.1 Conjugated polymer nanoparticles

NPs prepared from conjugated polymers represent an established class of nanomaterials with exceptional photophysical properties. Owing to their large molar extinction coefficients, tunable emission, high fluorescence quantum yield, and photostability, conjugated polymer nanoparticles (CPNs) or polymer dots (Pdots, particle size <30 nm) are versatile tools for various applications.^43,83,84^ Because of their biocompatibility and inherent optical properties, CPNs are widely used in single photon,^85–93^ multiphoton,^94,95^ photoacoustic^96,97^ and super-resolution^24^ imaging applications. CPNs have been used to develop various ultrasensitive biosensors^98–100^ and they were successfully used for cell labelling,^56,101^ flow cytometry^102^ and theranostics applications.^103–105^ We recommend the readers several excellent reviews focused on design and applications of CPNs and Pdots.^83,84,99,106–108^ Below we analyse the brightness of typical examples of CPNs.

**Fig. 4:**
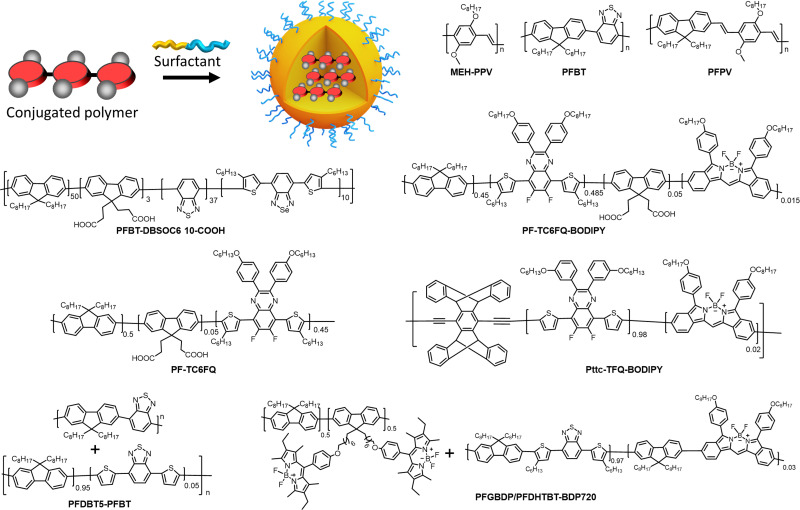
Fluorescent NPs based on conjugated polymers: preparation and their chemical structures.

In the seminal work, McNeill and co-workers prepared CPNs based on PFPV, polyfluorene benzothiadiazole (PFBT) and MEH-PPV, absorbing and emitting in the visible (Fig. 4).^55^ It was shown that the small size of NPs was crucial to achieve high QY values, therefore, the focus was made on small CPNs of 15 nm diameter. MEH-PPV emitting in the red region showed the weakest QY values (1%), but high extinction coefficient of particles of 1.2 × 10^8^ M^−1^ cm^−1^. As a result, the brightness of these NPs according to eqn (5) was 1.2 × 10^6^ M^−1^ cm^−1^ and per-volume brightness (*B*_V_) according to eqn (9) of 650 M^−1^ cm^−1^ nm^−3^ (Table 1). PFPV and PFBT operating in the green and orange region, respectively, showed higher QY around 7 and 8%, respectively. Their corresponding brightness reached values of 1.2 × 10^7^ and 5.1 × 10^6^ M^−1^ cm^−1^, with *B*_V_ of 6500 and 2900 M^−1^ cm^−1^ nm^−3^. Later on, PFBT NPs were further improved by encapsulation into the block copolymer PS–PEG–COOH (Fig. 4).^56^ PFBT dots had small size of 10 nm and high extinction coefficient (1 × 10^7^ M^−1^ cm^−1^) and QY of 30%. The achieved brightness was 3× 10^6^ M^−1^ cm^−1^, while *B*_V_ reached 5700 M^−1^ cm^−1^ nm^−3^ (Table 1). According to single-particle measurements by fluorescence microscopy, these NPs were 30-fold brighter than QD-565 (excitation at 488 nm), which positioned semiconductor polymer NPs as ultrabright nanomaterials.

With efforts from various research groups, good quantum yields and brightness were achieved for CPNs that absorb and emit in the visible region.^109,110^ However, CPNs that emit in the far-red to NIR region suffer from poor quantum yield due to the ACQ of large pi-conjugated polymer units. Probes with far-red to NIR emission are very useful for bioimaging because of low light-scattering and auto-fluorescence of live tissues in the NIR region, allowing imaging at higher penetration depth.^111^ Therefore, there is a significant interest in developing bright CPNs that emit in far-red to NIR window. Chen *et al.* developed dithienylbenzoselenadiazole (DBS) based NIR emitting donor–acceptor (D–A) type Pdots (PFBT-DBSOC6 10-COOH) (Fig. 4).^57^ DBS-based Pdots exhibited large extinction coefficient (7.4 × 10^7^ M^−1^ cm^−1^). The long-alkyl chains present on thiophene units reduced the close packing of DBS monomers in the Pdots, which in turn helped to maintain reasonably high quantum yields (15%). The resulting 29 nm DBS based Pdots had a brightness of 1.1 × 10^7^ M^−1^ cm^−1^ and *B*_V_ of 1758 M^−1^ cm^−1^ nm^−3^ (Table 1). These Pdots were 2 times brighter than Qdot 705 (excitation at 488 nm).

A promising direction in designing of NIR CPNs is to incorporate a powerful fluorophore BODIPY directly into the conjugation backbone, as exemplified by PF-TC6FQ-BODIPY (Fig. 4). The BODIPY unit serves as energy acceptor used at low molar ratio with respect to energy donor quinoxaline units, which allows to shift emission to the red and decrease self-quenching phenomenon for the donor units. The obtained 26 nm CPNs showed far-red to NIR emission (723 nm) with good QY (33%), with brightness of 1.7 × 10^7^ M^−1^ cm^−1^ and *B*_V_ of 1950^−1^ cm^−1^ nm^−3^ (Table 1).^58,59^ In order to further address the ACQ in NIR fluorescent Pdots, Chen and co-workers developed BODIPY-containing donor–acceptor conjugated polymers bearing AIE-active tetraphenylethene (TPE) unit and a bulky pentiptycene (pttc) unit (Pttc-TFQ-BODIPY). A control polymer containing polyfluorene donors conjugated to BODIPY based acceptor was used for comparison.^60^ The resulting 28 nm sized Pdots showed absorption in the visible region (490 nm) with remarkably high extinction coefficients (1 × 10^8^ M^−1^ cm^−1^) and emission in the NIR window (724 nm). Modification in the polymer backbone with bulky substituents significantly increased the QY of the resulting Pdots. Pdots derived from bulky Pttc-based polymer and tetraphenyl ethylene (TPE)-based polymer exhibited high QY of 51% and 37%, respectively, compared to those from the control polymer (7%). The *B*_V_ value of Pttc-TFQ-BODIPY Pdots was 4481 M^−1^ cm^−1^ nm^−3^ (Table 1), whereas single-molecule microscopy suggested that they were 5 times brighter than QD 705 when excited at 473 nm. The same group reported quinoxaline based semiconducting polymer dots PF-TC6FQ (Fig. 4).^61^ These 21 nm sized NPs showed far-red emission and high QY of 46% and *B*_V_ of 4961 M^−1^ cm^−1^ nm^−3^, whereas they were 8 times brighter than Qdot 655 (excitation at 488 nm).

Chiu and co-workers developed semiconducting polymer blend dots (PBdots) for *in vivo* tumor targeting. D–A type PBdots, PFDBT5–PFBT were prepared by PFBT polymer donor and deep red-emitting PFDBT5 polymer as acceptor (Fig. 4).^42^ The resulting 15 nm PBdots showed absorption (450 nm) and emission (650 nm) in the visible to far-red region. PBdots had large molar extinction coefficient (*ε* = 3.7 × 10^7^ M^−1^ cm^−1^) and excellent quantum yield (QY = 56%), which is so far one of the highest QY reported for CPNs. These PBdots exhibited an average *B*_V_ of 9500 M^−1^ cm^−1^ nm^−3^. Single-particle imaging suggested that PBdots were 15 times brighter than Qdot 655 (excitation at 488 nm). Later on, Wu and Chiu groups reported ultrabright, narrow band, NIR emissive blend Pdots, PFGBDP/PFDHTBT-BDP720.^62^ These Pdots were prepared from D1/D2-A type conjugated polymers. D1-Polymer was constructed by grafting green emitting BODIPY to a polyfluorene (PFO) backbone and D2-A was constructed by introducing BODIPY720 acceptor into PFDHTBT conjugated polymer (Fig. 4). The resulting 31 nm Pdots showed absorption in the visible region (528 nm) and NIR emission (721 nm). These Pdots exhibited extremely high extinction coefficient of 4.2 × 10^8^ M^−1^ cm^−1^. Quantum yield of such Pdots was 40%, so that brightness and *B*_V_ reached very high values: 1.8 × 10^8^ M^−1^ cm^−1^ and 11 308 M^−1^ cm^−1^ nm^−3^, respectively (Table 1), the latter being the highest per-volume brightness reported for CPNs. Single-particle brightness measurements showed that Pdots PFGBDP/PFDHTBT-BDP720 were 83 times brighter than Qdot 705 when excited at 532 nm.

Overall, analysis of the brightness of CPNs from various literature reports suggest that along with fine tuning of the particle size, careful molecular engineering of the polymers is particularly important to achieve high brightness. On the one hand, optimum conjugation is necessary to obtain higher extinction coefficients. On the other hand, bulky non-planar substituents on the polymer chain are necessary to improve the quantum yield by preventing ACQ. Donor–acceptor type conjugated polymers with an optimized acceptor ratio is so far the most successful design to obtain bright longer wavelength emissive CPNs. An emerging direction is reaching NIR-II region with CPNs, which can further boost tissue penetration depth,^105,112^ however, brightness of these systems still needs improvement. An important tendency is that for similar polymers, CPNs of smaller size showed higher QY values and thus higher per-volume brightness (Table 1).^55^ Larger sizes probably increase the chances of the fluorescence quenching by a fraction of the polymer present in the dark state within NPs. Therefore, design of bright CPNs operating in red to NIR regions will require both optimal small size and careful molecular engineering, which includes strong conjugation, bulky side groups, and eventually the use of donor–acceptor configuration.

### 4.2. Aggregation-induced emission nanoparticles

The AIE concept is particularly suitable to prevent ACQ in nanomaterials, because the dyes are specifically designed to be highly emissive in the solid state. Since the first report on AIE materials by Tang *et al.*,^44^ tremendous research efforts were done to develop bright fluorescent (nano)materials with varied optical properties and broad range of applications, such as organic light emitting diodes (OLEDs),^113,114^ sensing,^115,116^ single photon,^117^ multiphoton,^65,118^ NIR-II bioimaging,^68,119,120^ image-guided surgery^67,121^ and phototherapy.^122–125^ There are several excellent reviews on the mechanism and applications of this remarkable phenomenon.^126–129^ In this tutorial review, we only highlight the strategies focused on improving the brightness of AIE NPs of different colour.

**Fig. 5:**
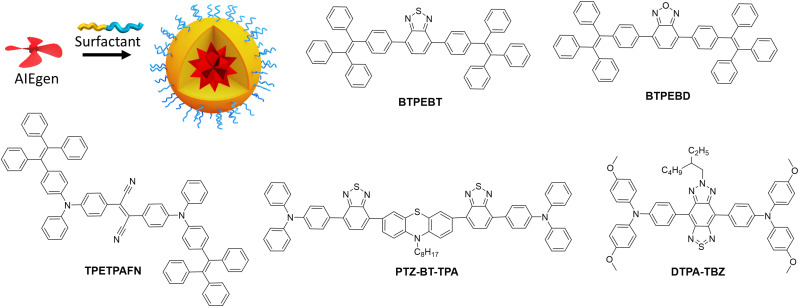
Scheme of preparation of AIE NPs and chemical structure of AIEgens.

In an early study, Tang and Liu groups reported red emitting NPs (AIE dots) based on dicyano-substituted stilbene bearing tetraphenylethylene (TPE) and triphenylamino (TPA) units (TPETPAFN, Fig. 5), which were coated with the lipid–PEG conjugate DSPE–PEG and functionalised with a cell penetrating peptide.^63^ The obtained NPs displayed an average size of 33 nm, quantum yield of 25%^63^ and extinction coefficient of 4.2 × 10^7^ M^−1^. Having brightness of 1.1 × 10^7^ M^−1^ cm^−1^ (*B*_V_ = 560 cm^−1^ nm^−3^, Table 1), these AIE dots were 10 times brighter than QD 655 in single-particle measurements (488 nm excitation). The same teams also developed AIE dots based on 4,7-bis[4-(1,2,2-triphenylvinyl)phenyl]benzo-2,1,3-thiadiazole (BTPEBT, Fig. 5) encapsulated in a DSPE–PEG_2000_ shell.^64^ The resulting 30 nm sized AIE-dots showed absorption with large molar extinction coefficient (5.9 × 10^7^ M^−1^ cm^−1^) and green emission high quantum yield (QY = 63%). Their brightness reached 3.5 × 10^7^ M^−1^ cm^−1^ and *B*_V_ was 1980 M^−1^ cm^−1^ nm^−3^. AIE dots based on BTPEBT coated with DSPE–PEG-coating (BTPEBT-V2, Table 1) were also developed for two-photon imaging application^65^ with slightly smaller particle size (29 nm) and improved *B*_V_ (2870 M^−1^ cm^−1^ nm^−3^).

Liu and co-workers further developed AIE dots based on BTPEBD (4,7-bis[4-(1,2,2-triphenylvinyl)phenyl]benzoxadiazole),^66^ where benzothiadiazole of BTPEBT was replaced with a benzoxadiazole unit (Fig. 5). The orange emissive BTPEBD-AIE nanoparticles had an average size of 32 nm and exceptionally high QY of 90%. BTPEBD AIE dots showed high brightness 5.3 × 10^7^ M^−1^ cm^−1^ and their *B*_V_ reached up to 3100 M^−1^ cm^−1^ nm^−3^ (Table 1).

Tang and co-workers recently reported the AIEgen PTZ–BT–TPA presenting both planar and twisted units based on phenathiazine (PTZ), benzothiadiazole (BT) and triphenylamine (TPA).^67^ The resulting PTZ–BT–TPA dye had high extinction coefficient (6.24 × 10^4^ M^−1^ cm^−1^) and AIE properties. The NPs prepared from PTZ–BT–TPA encapsulated in DSPE–PEG_2000_ had an average diameter of 100 nm and QY of 39%. PTZ–BT–TPA particles had very high brightness of 2.4 × 10^9^ M^−1^ cm^−1^ and their *B*_V_ reached as high as 4600 M^−1^ cm^−1^ nm^−3^ (Table 1), which is one of the highest values reported for AIE NPs to date. Meng and co-workers reported D–A–D type AIE dots that emit in the NIR-II window (900–1700 nm), attractive due to higher penetration depth for *in vivo* imaging applications.^68^ The dye was designed by coupling triphenylamine (TPA) donor units on either side of the thiadiazolobenzotriazole (TBZ) acceptor core (Fig. 5). The 50 nm AIE dots prepared from DTPA–TBZ displayed long-wavelength absorption (652 nm) and NIR-II emission (929 nm). DTPA–TBZ AIE dots had a quantum yield of 11% and brightness of 4.0 × 10^7^ M^−1^ cm^−1^. Even though their *B*_V_ was lower (614 M^−1^ cm^−1^ nm^−3^) compared to other AIE systems, it is significant considering emission in the NIR-II window.

Overall, the AIE concept enables preparation of bright NPs with high quantum yields and high dye concentration, because the NPs core is generally composed of pure dye. Nevertheless, their brightness is currently limited by molar extinction coefficients of AIEgens, which are smaller compared to conventional dyes. On the one hand, the twisted molecular structures of AIEgens help them to achieve good quantum yield by reducing ACQ, on the other hand, their twisted non-planar architecture decreases the pi-conjugation and thus the molar extinction coefficients. Thus, it will be important to find a balance between the conjugation and non-planar structure to improve both the quantum yield and extinction coefficient of AIE nanomaterials. Recent report by Tang co-workers on PTZ–BT–TPA AIEgen^67^ showed a path forward to achieve this balance. Moreover, the future efforts will be devoted to the development of bright AIEgens in NIR-I and NIR-II windows.

### 4.3. Nanomaterials derived from conventional organic dyes

The opportunity to assemble nanomaterials from typical fluorescent dyes looks particularly attractive. Indeed, a great variety of fluorescent dyes with desired optical properties, *e.g.* BODIPYs, perylene diimides (PDIs), rhodamines, cyanines, *etc.*, are available and chemically assessable. Moreover, they exhibit high molar extinction coefficients and fluorescence QYs, which makes them bright single-molecule emitters with *B*_V_ values reaching 10^5^ M^−1^ cm^−1^ nm^−3^ for rhodamine 6G (Table 1). However, highly-emissive nanomaterials cannot be obtained straight from conventional fluorescent dyes, because of the above-mentioned phenomenon of ACQ. Indeed, at high loading within a particle matrix (above 1 wt%) most of these dyes undergo clusterization and strong self-quenching. Moreover, non-covalently dye-loaded NPs tend to release significant amounts of encapsulated dyes leading to pronounced fluorescence background. Therefore, preparation of bright NPs from conventional dyes requires addressing simultaneously the problems of ACQ and dye leakage. Conceptually, methods to prevent ACQ in solid state are different for neutral and charged dyes, therefore hereafter they are presented separately.

#### 4.3.1. Neutral dyes

A large variety of dyes are uncharged (neutral), in particular, BODIPY, PDIs, squaraine, Nile Red and some others. Absence of charge makes them intrinsically hydrophobic, which is convenient for their encapsulation into a hydrophobic matrix of polymeric and lipidic NPs. Unfortunately, these dyes present strong ACQ already around 1 wt% of dye loading, limiting brightness of the obtained materials. For example, Resch-Genger and co-workers demonstrated that the hydrophobic dye Nile Red can be encapsulated into 100 nm crosslinked polystyrene (PS) NPs to reach maximum brightness at 0.8 wt%.^69^ The QY values decreased from 76% (at 0.05 wt% loading) to 23% (at 0.7 wt%), allowing to reach high mean brightness of individual 100 nm particles of 7 × 10^7^ M^−1^ cm^−1^, but relatively low *B*_V_ of 134 M^−1^ m^−1^ nm^−3^.

To prevent ACQ in neutral dyes, the most common approach is to introduce bulky side groups.^46–48^ In contrast to AIE approach, it exploits “classical” flat dyes, and their pi-stacking is prevented by introduced out-of-plane bulky groups.^46,47,130,131^ Importantly, these bulky side groups also decrease the tendency of dyes to crystallize and increase their hydrophobicity, both favouring preparation of NPs. This method was effectively applied in case two dye families: BODIPYs and PDIs, which are presented below.

**Fig. 6:**
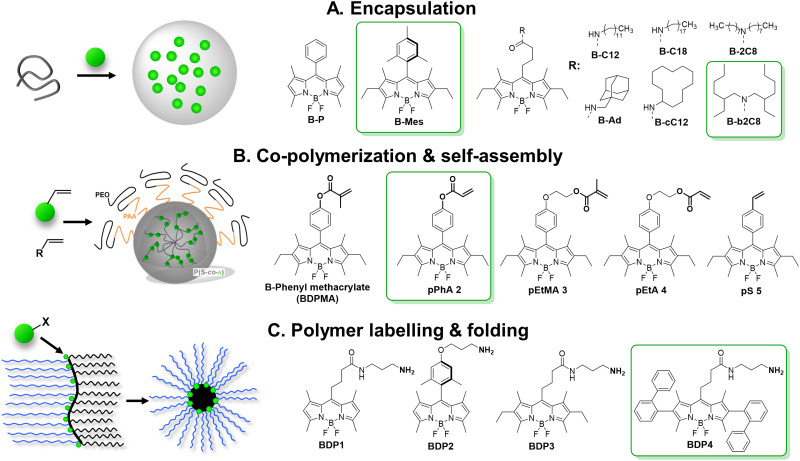
Schemes of preparation of fluorescent NPs based on BODIPY derivatives. Reproduced with permission from ref. 72 and 73. Copyright American Chemical Society.

BODIPY are bright organic dyes typically with high QY close to 1.0 and extinction coefficient above 70 000 cm^−1^ M^−1^. However, their flat chromophore core favours ACQ in solid state. In some early works, it was shown that sterically hindered bulky substituents introduced to BODIPY core prevented ACQ and yielded materials emissive in the solid-state.^132,133^ The classical example of sterically hindered dye is Mes-BODIPY, which was encapsulated into cross-linked polystyrene NPs (16 nm) by swelling in dichloromethane followed by evaporation.^71^ At 76 dyes loaded per particle, the QY remained high (77%) and achieved brightness of NPs was 5.1 × 10^6^ M^−1^ cm^−1^ with *B*_V_ of 2300 M^−1^ cm^−1^ nm^−3^. We compared Mes-BODIPY with a series of hydrophobic BODIPY derivatives (Fig. 6A) after their encapsulation into PLGA nanoparticles, prepared by nanoprecipitation.^75^ BODIPY with *meso*-alkyl substituents were systematically less resistant to ACQ (QY = 10–20% *vs.* 50% for Meso-BODIPY at 50 mM loading), probably due to their planar structure. On the other hand, their most hydrophobic analogues were stable against dye leakage from NPs in aqueous media with foetal bovine serum or being internalized into live cell, probably because they were better encapsulated inside the hydrophobic core of NPs.

A fruitful strategy to control the dye ACQ and encapsulation is to covalently graft dyes to the polymer backbone. Clavier with co-workers synthesized a reactive analogue of sterically hindered BODIPY and co-polymerized them in miniemulsion to yield NPs (Fig. 6B).^72^ The synthesis relied on polymerization-induced self-assembly (PISA) – folding of growing chains of amphiphilic PEGylated polyacrylate–polystyrene block copolymer forming core–shell nanoparticles. All five BODIPY monomers demonstrated excellent inclusion rate (from 0.92 to 0.98) targeting 3 dyes per a chain (15–20 kDa, ∼8 wt% dye loading). Up to 5000 BODIPY acrylates per particle were loaded for 60–90 nm size of NPs, while reserving relatively high QY values (35%). By theoretical calculation they were 200–2000 brighter NPs than quantum dots (1.4 × 10^8^ M^−1^ cm^−1^) with *B*_V_ of 470 M^−1^ cm^−1^ nm^−3^. Later on, the same group showed that brightness could be further increased by inclusion of higher dye content, but QY gradually decreased presumably due to ACQ.^134^

We followed a quite different strategy, where sterically hindered BODIPY dyes were covalently attached to an amphiphilic polymer capable to form single-polymer NPs of small size (Fig. 6C).^73^ In this case, poly(maleic anhydride-*alt*-1-octadecene) (PMAO, 30 kDa) was modified in two steps with BODIPY dye and PEG groups. In water this polymer folded into small single-chain NPs, bearing the dye in the hydrophobic core and PEG groups on the surface. Among tested BODIPYs, BDP4 bearing two diphenyl substituents was the most resistant to ACQ: no hypochromism or absorbance band broadening or significant H-aggregate band appeared upon increasing dye content from 2 to 50 mol%, compared to other dyes (BDP1–3). BDP4–PEG(1000)–PMAO NPs showed QY of 60% at 50 mol% dye content (*i.e.* 18 wt%) and high 2.5 × 10^6^ M^−1^ cm^−1^ brightness for monomolecular nanoparticles of 14 nm in diameter, with *B*_V_ of 1740 M^−1^ cm^−1^ nm^−3^ (Table 1).^73^ Single-particle measurements showed that these NPs were 5-fold brighter than QD-585 (at 532 nm excitation).

**Fig. 7:**
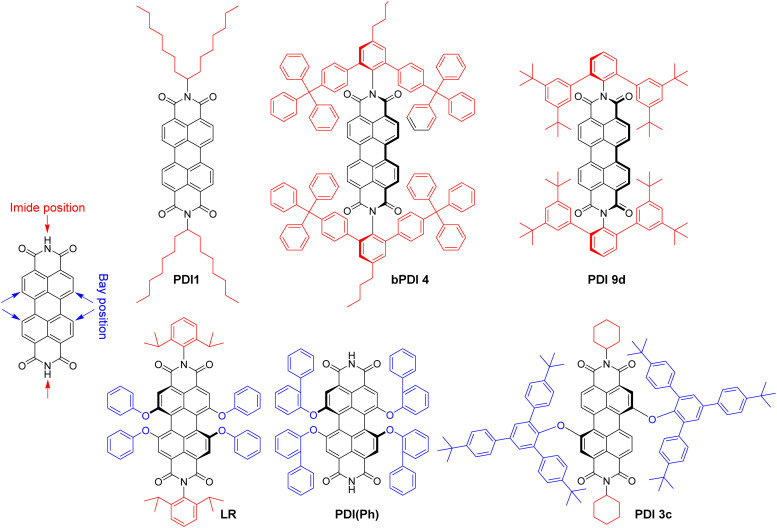
Molecular structure of perylene diimides (PDI) with bulky substituents.

Polyaromatic imides are a unique class of organic emitters, characterized by high brightness and phenomenal photostability. On the other hand, their poor solubility and tendency to aggregation due to strong pi–pi stacking makes them particularly prone to ACQ. For example, perylendiimide (PDI) forms non-emissive (H-aggregate) and emissive red-shifted (J-aggregate or excimer) species in solid state depending on substituents in the bay and imide sides (Fig. 7), as summarized by Würthner and co-workers in a recent review.^135^ However, the vast majority of the examples deals with bulk materials without formulation of NPs. Wong and co-authors designed PDI insulated with branched aromatic substituents at imide positions.^46^ Compound bPDI4 bearing bulkiest groups (Fig. 7), exhibited QY of 29% and *B*_V_ of 8700 M^−1^ cm^−1^ nm^−3^ in solid state, whereas by inclusion into PMMA thick film at a relatively high (120 mM) concentration, QY raised up to 70%, but the *B*_V_ value decreased twice (3700 M^−1^ cm^−1^ nm^−3^). Würthner and co-workers reported PBI9d with highly sterically hindered substituent, which gave QY of 17% and *B*_V_ of 8300 M^−1^ cm^−1^ nm^−3^.^136^ Even more efficient is shielding of PDI dye with bulky substituents at the bay region. For example, PDI(Ph) with four *ortho*-phenyl-phenoxy groups exhibited highest QY in crystals (59%) with dye extinction coefficient of 42 300 M^−1^ cm^−1^ and *B*_V_ of ∼18 000 M^−1^ cm^−1^ nm^−3^ (calculated with parameters of X-ray crystal structure).^137^ Even higher brightness could be achieved with *N*,*N*′-dicyclohexylperylene diimide sterically fully enwrapped with two 2,4,6-tris(4-*tert*-butylphenyl)phenoxy groups at bay positions (PBI3c, Fig. 7).^47^ Quantum yield of PBI-3c in monocrystals reaches 84% along with *B*_V_ of almost 30 000 M^−1^ cm^−1^ nm^−3^. However, the possibility to transform these ultra-bright bulk materials into bright fluorescent NPs has not been demonstrated do date.

The examples of incorporation of PDI dyes into nanoparticles are very rare, and limited to derivatives with much smaller side groups. In an early study, Li and co-workers incorporated PDI derivative bearing chlorines at the bay regions (PDI-Cl) into polymer chains by co-polymerization.^74^ 40 nm cross-linked polymer NPs were obtained with PDI-Cl dye loading of 2.4 wt% and QY value of 50%. The achieved brightness and *B*_V_ values were 1.0 × 10^7^ M^−1^ cm^−1^ and 310 M^−1^ cm^−1^ nm^−3^, respectively. Single-particle measurements revealed brightness equivalent to 50–220 PDI dye monomers. We studied the role of bulky substituent in the bay and imide regions for the formulation of fluorescent polymeric nanoparticles based on biodegradable polymer PLGA. We selected PDI-1 bearing branched alkyl groups in the imide part and Lumogen Red (LR, Fig. 7), substituted with bulky groups at the bay region, and encapsulated them into PLGA NPs by co-precipitation.^75^ PDI-1 showed a tendency to aggregation that increased from 0.02 to 1 wt% dye loading, observed as a broadening in the absorbance spectra and a rise in the red shifted excimer band in the emission spectra, whereas QY decreased from 67 to 31%. In contrast, LR showed better resistance to ACQ, displaying exclusively green emission of the molecular form up to 5 wt% loading and decrease in QY from 97% (0.02 wt%) to 47% (5 wt%). At 5 wt% LR loading, 38 nm NPs showed a brightness of 7.5 × 10^6^ M^−1^ cm^−1^ with *B*_V_ of 261 M^−1^ cm^−1^ cm^−3^, whereas in single-particle microscopy experiments they were 18-fold brighter than quantum dots emitting in the same range (QD-585 at 532 nm excitation).

Overall, neutral fluorescent dyes such as BODIPYs and PDIs, are bright mono-molecular emitters but tend to strong ACQ in solid state. This could be partially resolved by introduction of bulky and branched substituents, leading to a brighter fluorescence at high local dye concentration. Therefore, PDI derivatives reach record-breaking brightness per volume values in form of bulk solid materials. However, owing to their large flat pi-conjugated structure, they tend to crystallize, which makes it challenging to prepare them in form of NPs, either as pure dye NPs or encapsulated into polymer or lipid NPs. Nonetheless, appropriate functionalization of bulky and/or reactive groups allows fabrication of NPs of relatively high brightness. Further efforts should be directed to design of neutral dyes that keep the balance between the bulkiness of side groups and capacity to be encapsulated into organic NPs, while preserving their high brightness and other spectral characteristics in NPs.

#### 4.3.2. Ionic dyes

**Fig. 8:**
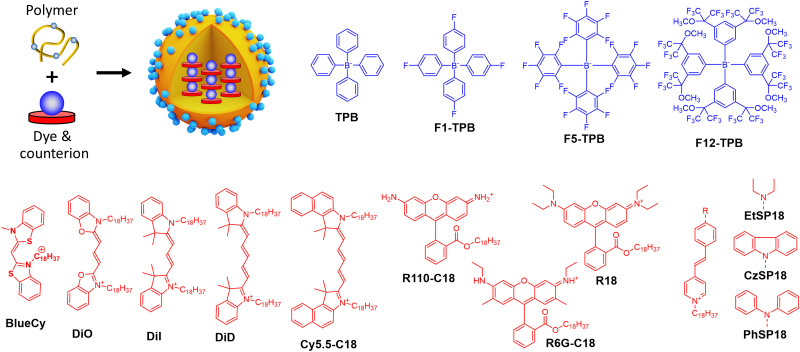
Dye-loaded polymeric NPs and examples of bulky counterions paired with cationic dyes for the NPs preparation.

Ionic dyes, represented by the large families of cyanine and rhodamine dyes, are among the brightest fluorophores developed to date.^1^ However, preparation of bright nanomaterials based on these dyes is particularly challenging, since methods based on bulky substituents are not so efficient for charged fluorophores. Moreover, their positive charge makes them more soluble in water, compared to neutral dyes, which further complicates formulation of NPs. Therefore, to formulate ionic dyes into lipid or polymer NPs, they are functionalized with hydrophobic groups, for examples with two octadecyl chains in DiO, DiI and DiD based on Cy2, Cy3 and Cy5 dyes, respectively (Fig. 8). Law and co-workers prepared PEGylated PLGA NPs of 70–90 nm size, encapsulated with 0.05–3 wt% of DID.^70^ The NPs showed satisfactory quantum yields of 21% for DID at 0.5 wt% loading, whereas at higher loading QYs drastically decreased. Thereafter, the highest brightness was achieved at 0.72 wt% of DID content formulated in 66 nm NPs with brightness of 1.3 × 10^7^ M^−1^ cm^−1^ and *B*_V_ of 84 M^−1^ cm^−1^ nm^−3^ (Table 1). Similar to above mentioned neutral dyes, encapsulation of charged dyes into NPs with dye content above 1–5 wt% results in a strong ACQ.

Insulation of positively-charged fluorophores could be efficiently performed with organic counterions. Earlier works of Yao *et al.*^51^ and Warner and co-workers^52^ demonstrated that bulky hydrophobic anions decrease ACQ in pure ion pairs in solid state form. Previously, we described preparation of fluorescent organic NPs from pure dye salts of alkyl esters of rhodamine B with different tetraphenylborate counterions (TPB, F1-TPB, F5-TPB, F12-TPB, Fig. 8) by nanoprecipitation.^76^ The increase in the alkyl ester chain length as well as higher fluorination level and bulkiness of the tetraphenylborate counterions significantly improved fluorescence quantum yield and stability of the obtained NPs. Thus, *O*-dodecyl rhodamine B (R12) with F5-TPB and F12-TPB NPs gave 14 and 19 nm NPs, respectively, with quantum yields of 40 and 60% respectively. Theoretical calculation based on eqn (5) gave brightness (*B*) values of 2.7 × 10^7^ M^−1^ cm^−1^ and 6.8 × 10^7^ M^−1^ cm^−1^ for R12/F5-TPB and R12/F12-TPB NPs, respectively, while eqn (11) gave impressive *B*_V_ values: 18 700 and 19 100 M^−1^ cm^−1^ nm^−3^, respectively (Table 1). According to FCS measurements, the experimental single-particle brightness of R12/F12-TPB NPs reached 2.4 × 10^7^ M^−1^ cm^−1^, which was equivalent to 540 single rhodamines or 45 QDs-585. However, they were not sufficiently stable in cells undergoing dye release, even though NPs with more fluorinated counterions were more stable.

To formulate stable dye-loaded NPs, we proposed to pair cationic dyes with bulky hydrophobic counterions and encapsulate them into the matrix of polymeric NPs by nanoprecipitation. In our first report, octadecyl rhodamine B (R18) paired with F5-TPB was encapsulated at 5 wt% (*vs.* polymer) into 40 nm PLGA NPs (Fig. 8). Owing to a QY of 21%, their estimated brightness reached 1.8 × 10^7^ M^−1^ cm^−1^ with relatively low *B*_V_ values of 600 M^−1^ cm^−1^ nm^−3^ (Table 1).^33^ Single-particle microscopy suggested that the single-particle brightness corresponded to 6 QDs-585 at 532 nm excitation and revealed neatly complete ON/OFF particle blinking, caused by fast dye–dye energy transfer.^33^ The same dye was then encapsulated at 5 wt% into much smaller (15 nm) NPs built of sulfonated PMMA derivative, yielding NPs with improved *B*_V_ of 1600 M^−1^ cm^−1^ nm^−3^,^77^ due to a high fluorescence quantum yield (60%) in PMMA-based matrix. They were 10-fold brighter than QD-585 at 532 nm excitation. Further studies showed that more hydrophobic PMMA matrix ensured systematically higher quantum yields compared to PLGA, which could be explained by more even distribution of the dye without clustering inside the particle core. In this study, for 30 wt% loading *vs.* polymer (23 wt% of total NP mass) the brightness values reached 8.9 × 10^7^ M^−1^ cm^−1^ for 34 nm PMMA-MA NPs, which means the *B*_V_ values of 4730 M^−1^ cm^−1^ nm^−3^ (Table 1).^24^ According to single-particle microscopy, PMMA-MA NPs reached brightness equivalent to 100 QDs-585 (at 532 nm excitation), while their blinking was largely supressed compared to PLGA NPs. Moreover, 40 nm PMMA-based NPs loaded with 30 wt% of R18/F5-TPB *vs.* polymer (23 wt% of total NP mass) were decorated with DNA, yielding DNA nanoprobes with 46% QY, corresponding to a brightness of 1.9 × 10^8^ M^−1^ cm^−1^ and *B*_V_ of 5790 M^−1^ cm^−1^ nm^−3^ (Table 1).^35^ Single-particle measurements showed that these NPs were equivalent to 100 QD-605 excited at 488 nm. Next, replacing the PMMA matrix with the more hydrophobic PEMA enabled encapsulation of 50 wt% (33 wt% of total NP mass) of the dye into a smaller NPs’ core of 20 nm functionalized with DNA.^36^ These NPs displayed even higher QY of 52% than PMMA-MA NPs and a brightness of 3.8 × 10^7^ M^−1^ cm^−1^, whereas the *B*_V_ value reached 9480 M^−1^ cm^−1^ nm^−3^ (Table 1). Single-molecule microscopy showed that these 20 nm NPs were 87-fold brighter than QDs-605 measured at 550 nm excitation and they showed more stable emission (lower NP blinking) than their PMMA-based analogues. Remarkably, high fluorophore content in polymeric NPs enables highly efficient FRET to few acceptor fluorophores on the surface of NPs, leading also to increase in the overall QY.^35,36^

Thereafter, the concept of bulky tetraphenylborate counterion gained versatility towards different classes of fluorophores. Other hydrophobic rhodamine and cyanine dyes demonstrated compatibility with tetraphenylborates for encapsulation into a polymer matrix in order to broaden spectral operating range of fluorescent NPs (Fig. 8 and Table 1). For example, BlueCy/F5-TPB emitting in blue spectral range at 12 wt% in 40 nm PMMA-MA NPs enhanced drastically QY up to 17% compared to 0.1–0.3% in solution.^78^ Brightness of the NPs was ∼70-fold higher than for QD-525 at 470 nm excitation, with brightness of 2.3 × 10^7^ M^−1^ cm^−1^ and *B*_V_ of 690 M^−1^ cm^−1^ nm^−3^ (Table 1). Octadecyl-substituted analogue of rhodamine 6G was mostly efficient with F12-TPB counterion in 44 nm PMMA NPs at 250 mM (*vs.* polymer) loading with QY of 23%, brightness of 1.1 × 10^8^ M^−1^ cm^−1^ and *B*_V_ of 2490 M^−1^ cm^−1^ nm^−3^.^79^ F12-TPB was also found to be particularly efficient to prevent ACQ of cyanine dyes in NPs based on PLGA^138^ and PMMA^54^ polymers. For example, cyanine 5 dye DiD loaded at 30 wt% (23% of total NP mass) into 16 nm PEMA-based NPs showed QY of 42%, which corresponded to a particle brightness of 9.7 × 10^6^ M^−1^ cm^−1^ and *B*_V_ of 4530 M^−1^ cm^−1^ nm^−3^.^54^ Single-particle measurements showed that these small NPs were 22-fold brighter than QD-705 at 640 nm excitation. Bulky counterions can also enhance QY of cationic AIE dyes.^139^ Previously, we studied a styryl pyridinium dye family: EtSP18, CzSP18, and PhSP18 (Fig. 8), which presented or not AIE properties.^80^ Among them EtSP18, which was not emissive in solution or solid state, lighted up only in the presence of bulky hydrophobic counterions. We named this phenomenon “ionic AIE”, due to the key role of bulky counterions that light up non-emissive dyes in the solid state. Inside polymeric NPs these ion pairs exhibited efficient fluorescence: PhSP18/F5-TPB encapsulated in 40 nm PMMA NPs at 40 wt% dye loading (29% of total NP mass) showed 40% QY and fluorescence brightness of 1.2 × 10^8^ M^−1^ cm^−1^ with *B*_V_ value of 3840 M^−1^ cm^−1^ nm^−3^, which was equivalent to 50 QD-605 at 488 nm excitation in the single-particle microscopy measurements.^80^

Ionic dye insulation with bulky counterions was also found applicable to lipid nanoemulsions. Dioctadecyl Cy3 dye – DiI with TPB counterion was loaded into labrafac nanoemulsion up to 8 wt%.^81^ The increase in the dye loading from 0.1 to 8 wt% *vs.* oil core, decreased quantum yield by only 3.5-fold to 14%, which led to 87 nm nanoemulsion droplets encapsulating ≈12 000 dyes per droplet. This value corresponded to a brightness of 2.5 × 10^8^ M^−1^ cm^−1^, close to that obtained experimentally (8.0 × 10^7^ M^−1^ cm^−1^) by single-particle microscopy with reference particles (FluoSpheres) of known brightness. However, the *B*_V_ value of these lipid NPs (725 M^−1^ cm^−1^ nm^−3^) was significantly lower compared to the polymer NPs, which can be explained by both lower QY and lower dye loading.

Overall, ionic dye insulation with bulky hydrophobic counterions appears as a universal concept to prevent ACQ in charged dyes of different nature. Counterions play here multiple roles: (i) preventing dyes from H-aggregation, related to pi-stacking; (ii) enhance dye encapsulation into polymeric and lipid NPs because of high hydrophobicity of the obtained ion pair; and (iii) providing highly rigid surrounding for a dye.

**Fig. 9:**
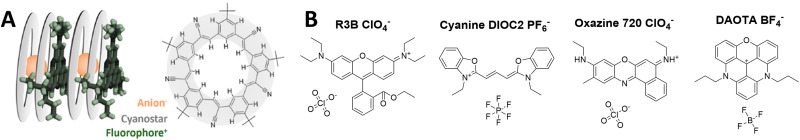
(A) Visual representation of SMILES concept and cyanostar microcyte and (B) fluorescent dyes insulated with anion-2-cyanostar complex. Panel A reproduced with permission from ref. 140. Copyright American Chemical Society.

Recently, the teams of Laursen and Flood presented an analogous concept called SMILES (*i.e.* small-molecule ionic isolation lattices), which used a supramolecular counterion complex built of the small inorganic anions (BF_4_^−^, PF_6_^−^ and ClO_4_^−^) with cyanostar – a planar conjugated macrocycle (Fig. 9A).^141^ Two cyanostars form a cage complex with one anion such as ClO_4_^−^, PF_6_^−^, BF_4_^−^. At the same time, this planar counterion complex alternates with dyes in stacked columns in crystal structure with mean dye–dye distance around 15 Å. This splitting distance prevents to some extent ACQ through the H-aggregation. This approach was successfully applied to a variety of cationic dyes, including triangulenium dyes (Fig. 9B), rhodamines and cyanines in form of bulk materials, such as films and crystals. The highest *B*_V_ values were obtained with rhodamine 3B perchlorate and cyanine 3 hexafluorophosphate dyes 9700 M^−1^ cm^−1^ nm^−3^ (QY 29%) and 11 800 M^−1^ cm^−1^ nm^−3^ (QY 25%), respectively. Later work showed that using energy transfer within mixed rhodamine R3B – cyanine DIOC2-based FRET SMILES, even higher quantum yields can be achieved (65%), reaching an impressive *B*_V_ value of 32 200 M^−1^ cm^−1^ nm^−3^ in bulk materials.^140^ The same groups used the SMILES concept with rhodamine derivative R12 in order to obtain small NPs of 16 nm size stabilized with DSPE–PEG lipid.^82^ The obtained NPs contained 400 dyes exhibiting QY of 30%, which corresponded to brightness of 1.5 × 10^7^ M^−1^ cm^−1^ and *B*_V_ of 5000 M^−1^ cm^−1^ nm^−3^ (Table 1). In the single-molecule microscopy, they were 20-fold brighter than the reference NPs 40 nm FluoSpheres®.

## 5. Brightness and biological applications

Owing to their unique optical properties, multifunctional surface chemistry and nanometric size, fluorescent NPs cover an immense range of biological applications.^4^ On the one hand, fluorescent organic NPs are advanced optical probes in biological media, cells and live animals, allowing high-contrast, high-resolution,^5,22,142^ and multimodal (*e.g.* photoacoustic) biological/biomedical imaging^13,106^ as well as theranostics applications involving phototherapy.^8,12,124,129^ On the other hand, they are powerful platforms for building sensors for small molecules and biomolecules^36,83,103^ as well as temperature and mechanical forces.^19^ Here, we will focus on some examples of biosensing and bioimaging applications where brightness of fluorescent organic NPs plays a particularly important role.

### 5.1. Bioimaging applications

**Fig. 10:**
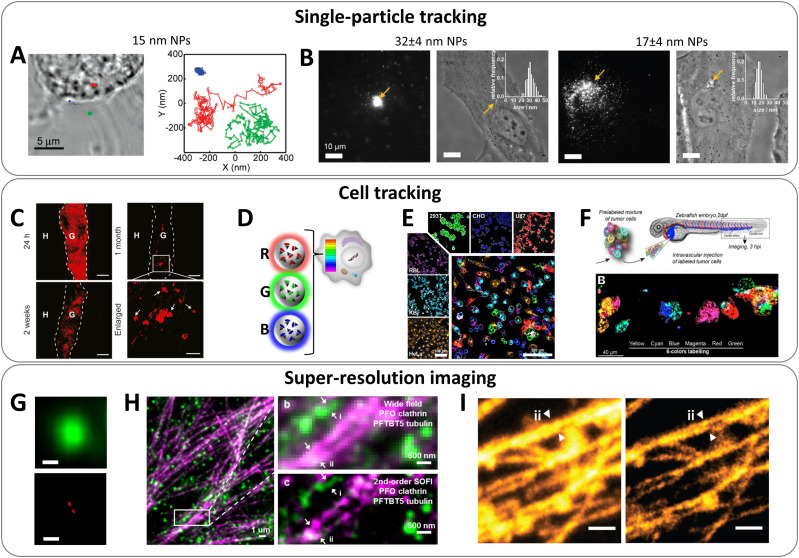
Application of bright organic NPs for bioimaging. (A) Tracking 15 nm PFBT Pdots in macrophage-like J774 cells. Left: Transmission image of a fixed cell. The colour marks indicate the locations of NPs: particle bound to the membrane (blue), outside the cell (green), and in the cell interior (red). Right: The trajectories for the three particles. Reproduced with permission from ref. 145. Copyright American Chemical Society (B) epi-fluorescence and phase-contrast images of HeLa cell microinjected with 32 and 17 nm dye-loaded PMMA-based NPs. Injection points are indicated by arrows. Scale bars, 10 μm. Insets show distributions of particle sizes obtained by TEM. NPs were loaded with 10 wt% of R18/F5-TPB and coated with Tween 80. Reproduced with permission from ref. 25. Copyright John Wiley and Sons. (C) Tracking transplanted neurons *in vivo*. D16 hESC-derived neurons were labelled with 30 nm TPETPAFN AIE-NPs for 24 h prior to transplantation into mouse brain striatum. Brain tissues were collected 24 h, 2 weeks, and 1 month post-transplantation. Scale bar: 100 μm, enlarged panel scale bar: 50 μm. Reproduced with permission from ref. 149. Copyright Elsevier. (D) Principle of cell barcoding by 40 nm dye-loaded polymeric NPs of three different colours: blue, green and red loaded with DiO/F12-TPB, DiI/F12-TPB and DiD/F12-TPB, respectively. (E) Tracking multiple RGB barcoded cell populations. The large micrograph shows a confocal image six cell types (HeLa, KB, 293T, U87, RBL, and CHO) mixed and co-cultured for 24 h. Each cell type was labelled with an RGB barcode (orange, cyan, green, red, magenta, and blue, respectively), also shown separately in the smaller images. Images are superpositions of the three NP channels with identical settings and of the membrane channel in grey. Scale bar is 100 μm. (F) Tracking RGB barcoded cancer cells in zebrafish embryo: six batches of D2A1 cells were labelled with fluorescent NPs generating RGB barcodes (green, red, blue, yellow, magenta, and cyan) and imaged 3 h post-injection. (9 D–F) – Reproduced with permission from ref. 138. Copyright John Wiley and Sons. (G) Top: Standard TIRF image of immobilized 40 nm dye-loaded NPs (PLGA, 5 wt% R18/F5-TPB); bottom: the same field after applying a super-localization procedure, showing capacity to resolve two particles (scale bar, 200 nm). Reproduced with permission from ref. 33. Copyright Springer Nature. (H) Dual-color superresolution (SOFI) imaging of subcellular structures labelled with small (10 and 13 nm) photoblinking Pdots. Left: Wide field imaging of clathrin coated pits labeled with PFO (green) Pdots and microtubule labelled with PFTBT5 (red) Pdots. Top right: Magnified region show in white box in left panel. Bottom right: SOFI image generated by analysing 500 frames of raw data from the wide-field image. Reproduced with permission from ref. 144. Copyright American Chemical Society. (I) STED imaging of the microtubule structures labeled using the AIE NPs (14–16 nm): confocal (left) and super-resolution STED (right) images of the microtubules. Reproduced with permission from ref. 150. Copyright John Wiley and Sons.

Brightness is essential to achieve high spatial and temporal resolution in fluorescence imaging. Single-particle tracking is probably the most demanding in terms of high brightness.^142^ It was originally developed for semiconductor QDs, for example, in tracking membrane receptors, which allowed tracking molecular diffusion with higher resolution and longer time compared to organic dye Cy3.^143^ Organic nanoparticles present attractive alternative to QDs for continuous single-particle tracking, because they can be much brighter and they generally do not blink (with some exceptions).^21,144^ McNeill and co-workers showed that high brightness of conjugated polymer NPs enabled fast tracking (50 Hz rate) with precision down to 1 nm and applied that to study complex diffusion behaviour in fixed cells (Fig. 10A).^145^ In a recent study using arginine modified CPNs, it was shown that particle entry inside the cells by endocytosis can be tracked at the single-particle level.^146^ Thus, Blanchard-Desce and co-workers showed that NPs assembled from push–pull dyes can be tracked with high precision inside live cells.^147^ Using single-particle tracking, we studied diffusion of dye-loaded polymeric NPs inside the cytosol as a function of their size. This study revealed critical size of NPs around 23 nm, below which NPs can diffuse freely in the cytosol (Fig. 10B).^25^ Further studies, in fixed and permeabilized cells suggested that small size (<20 nm) was also crucial for penetration of DNA-functionalized NPs inside the cells and detection of mRNA targets by fluorescence *in situ* hybridization (FISH), in line with earlier data on ultrasmall QDs.^54^ As the particle brightness decays as power of three of its diameter, a compromise should be made between small NPs size and the number of encapsulated dyes per particle. Based on the previous studies, the size around 10–20 nm could be considered as a good compromise. On the other hand, single-particle tracking in small animals requires much higher brightness, because of strong background and light scattering from the tissues. In the early studies, to track single NPs inside zebrafish embryo, we designed lipid NPs containing ∼10 000 cyanine dyes with bulky counterion (brightness was 2.5 × 10^8^ M^−1^ cm^−1^).^81^ One should note that in addition to high brightness, *in vivo* tracking requires compatibility with near-infrared imaging modalities, such as efficient two-photon absorption cross-section and/or near-infrared absorption/emission operating range. Thus, using polymeric matrix, we prepared ultrabright NPs of 74 nm dimeter by DLS and *B*_V_ of 4280 M^−1^ cm^−1^ nm^−3^, which according to two-photon FCS were 150-fold brighter than commercial Nile Red-loaded FluoSpheres. They enabled single-particle tracking directly in brain of live mice by two-photon microscopy and detection of NPs crossing the brain blood barrier.^148^

Cell tracking with NPs is another important application, especially given the growing interest in cell-based therapies. High brightness of NPs is crucial here, because cells can endocytose limited number of NPs and their tracking *in vivo* would require the strongest possible signal. Liu and co-workers designed 32–33 nm AIE dots functionalized with cell penetrating peptides in order to label cells. Due to their high brightness, stability and cell internalization, AIEs dots operating in green (BTPEBT) and red (TPETPAFN) regions allowed simultaneous discrimination of different populations of cancer cells both in culture medium and in animal organs.^63^ The red 30 nm AIE Dots (TPETPAFN) bearing TAT peptide were successfully applied for long-term labelling of neurons and their tracking in mouse brain striatum in various time points post-transplantation.^149^ These bright AIE dots allowed tracking neuronal grafts for up to 1 month (Fig. 10C). Using dye-loaded polymeric NP of different colour, which can be efficiently endocytosed by cells, we made long-term barcoding of cells and further tracking *in vitro* and *in vivo* on zebrafish (Fig. 10D–F).^138^ High brightness of NPs was important for the long-term tracking (over up to two weeks), because after each cell division the number of NPs per cell (initially ∼10 000) was divided by two. However, it still remains a challenge to extend the tracking time and achieve their tracking in small animals at sufficient depth.

High brightness of NPs is of particular interest for super-resolution imaging, because localization precision is intrinsically connected with the number of collected photons of the particle.^142^ However, this application is particularly challenging because NPs should be sufficiently small and present suitable optical properties. In particular, for single-molecule localization microscopy, it requires ON–OFF switching behaviour. Previously, we showed a phenomenon of collective blinking of >100 dyes within a dye-loaded polymeric particle, due to ultrafast dye-communication (Fig. 10G).^33^ Single-molecule localization microscopy (SMLM) imaging of single NPs revealed spots having width at half-maximum of 35 ± 7 nm, corresponding to their diameter, and possibility to resolve them at interparticle distances below the diffraction limit. Later on, Pdots were described based on PFO and PFTBT5, presenting small size (10 and 13 nm) and fluorescence blinking.^144^ They enabled two-colour SMLM (SOFI) imaging subcellular structures in cells with resolution down to 181 nm (Fig. 10H).^144^ Moreover, Wu, Sun and co-workers described photo-crosslinkable AIE NPs functionalized with streptavidin.^150^ The obtained NPs of three different colours, small size (14–16 nm) and high fluorescence quantum yields (24–39%) enabled STED-based super-resolution imaging of tubulin with resolution reaching 95 nm (Fig. 10I).

One should note that in this tutorial review, we focused only on a few examples of bioimaging applications of organic NPs, where brightness is particularly critical. One should mention other important applications, such as targeted imaging of tumours using NIR-I and NIR-II spectral regions^151,152^ as well as combination of bioimaging with photodynamic therapy,^112,153^ where CPNs and AIE NPs were particularly successful.

### 5.2. Biosensing applications

**Fig. 11:**
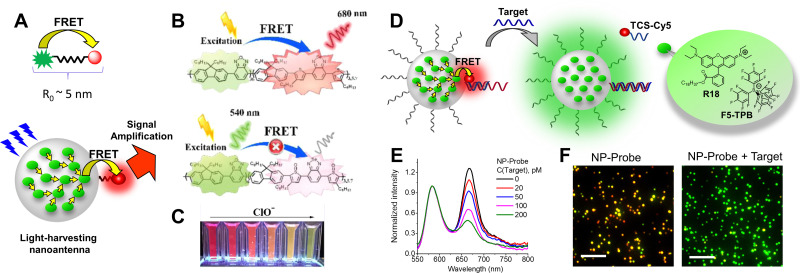
Applications of bright NPs for biosensing. (A) Top: FRET based molecular probe showing that the applications are limited to distances around the Förster radius. Bottom: FRET based nanoprobe where large number of donors are coupled due to excitation energy transfer (small yellow arrows) in order to ensure efficient transfer to a single acceptor. (B) Schematic Illustration of Pdot from donor–acceptor conjugated polymer. (C) Photographs of PFOBT36SeTBT5 Pdots with the addition of ClO^−^ taken under normal laboratory lighting and illumination with a UV light at 365 nm. (B and C) – Reproduced with permission from ref. 158. Copyright American Chemical Society. (D) DNA-functionalized dye-loaded polymeric nanoparticle (NP-Probe) for FRET-based detection of nucleic acids by stand displacement principle. R18 and its bulky counterion F5-TPB are also shown. (E) Fluorescence spectral response of NP-probe to the growing concentrations of the nucleic acid target. (F) Colour response of surface-immobilized NP-probe to the nucleic acid target (100 pM) at the single-particle level. (D–F) – Reproduced with permission from ref. 35. Copyright American Chemical Society.

NP brightness is also crucial for biological detection. The brightness defines the number of NPs that can be detected above the background and, therefore, the sensitivity of the assay. Indeed, NPs with 1000-fold higher brightness can be detected at 1000-fold lower concentration, typically from nM to pM range,^35^ compared to molecular probes, detectable in the μM–nM range. However, to work as probes for analytes, the sensing mechanism should be implemented that couples a molecular recognition event with the fluorescence response of the particle. In this respect, one of the most universal approaches is Förster resonance energy transfer (FRET), because it is highly sensitive to distances at the molecular scale around Förster radius of ∼5 nm (Fig. 11A).^154^ However, the general problem of fluorescent NPs is their limited FRET efficiency to a single acceptor, because their size is generally larger than the Förster radius. For example, in the case of QDs, multiple acceptors per particle are needed to achieve efficient FRET in biosensors.^155^ In this respect, conjugated polymer NPs are of particular interest for preparation of bright nanoprobes with efficient FRET for amplified biological sensing.^103^ Indeed, they exhibit high brightness and outstanding capacity to transfer energy due to fast excitation energy migration.^83^ A more recent example of bright organic NPs capable to undergo efficient FRET are dye-loaded NPs that use bulky hydrophobic counterions.^156^ At high dye loading the counterion not only prevent ACQ, but also ensures proper dye–dye spacing for ultrafast energy migration towards the FRET acceptor (Fig. 11A). As a result ∼10 000 dyes could efficiently transfer energy to a single dye acceptor, which generated giant antenna effect (signal amplification factor) of ∼1000, allowing detection of single molecules at ambient light-like conditions.^156^

The early examples of amplified sensing using light-harvesting and effective FRET in organic NPs were reported by Chiu and co-workers for conjugated polymer NPs (Fig. 11B).^157^ They developed CPNs of ∼23 nm size composed of donor and acceptor conjugated polymer units as probes for reactive oxygen and nitrogen species (ROS and RONS).^157^ The obtained nanoprobes exhibited strong acceptor emission in the intact form, but under action of ROS hypochlorite, the acceptor was bleached, which led to the enhancement of the donor emission (Fig. 11B and C). High brightness and operation in the far red region enabled ratiometric detection in hypochlorite with good limit of detection in solution (0.5 μM) as well as in cells and small animals.^157^ In another study, CPNs were functionalized with near-infrared dyes as FRET acceptors sensitive to ROS and nitrogen species. The obtained 78 nm nanoprobe generated a ratiometric response with a limit of detection for RONS at 10 nM and allowed their detection in cells and imaging inflammation in mice.^158^ More recently, FRET concept was applied for AIE NPs, using tetraphenylethylene derivative as a FRET donor and a hypochlorite-sensitive acceptor, allowing limit of detection of 105 nM and application to live cells.^159^ Based on bright dye-loaded polymeric NPs as light-harvesting nanoantenna undergoing efficient FRET to minimal amount of phosphorescent Pt–porphyrin, we developed a nanoprobe for amplified oxygen sensing with minimized cytotoxicity.^78^ Among the most challenging targets for amplified detection are nucleic acids, because they are present in biological samples at concentrations far below nanomolar. To this end, we grafted DNA to our light-harvesting nanoantenna based on 40 nm dye-loaded polymer NPs (Fig. 11D). Efficient energy transfer to few acceptors hybridized at the NPs surface enabled amplified detection of nucleic acids in solution and on surfaces (Fig. 11E and F) with the limit of detection of 0.5 and 10 pM, respectively.^35^ Further improved nanoprobe of 16 nm size with higher dye loading and per-volume brightness (9480 M^−1^ cm^−1^ nm^−3^) enabled detection of single nucleic acid copies that could switch emission of the whole particle after hybridization on its surface.^36^ One should note that in this case the limit of detection around 1 pM was defined by the kinetics of hybridization and not by the particle brightness.^36^ The concept was successfully applied for detection of microRNA cancer markers from cell extracts,^160^ showing compatibility with simple detection by a RGB camera of a smartphone.^79^ The performance of this system was dramatically enhanced by the recently found phenomenon of efficient long-distance FRET between dye-loaded NPs.^161^ In a recent report by Tian and co-workers, the light-harvesting nanoantenna concept was successfully realized for conjugated polymer NPs, resulting in the nanoprobes with a limit of detection of 1.7 pM for microRNA, which were further applied for RNA sensing at the single cell level.^109^

## 6. Conclusions and future perspectives

The field of fluorescent organic nanomaterials developed rapidly over the last decade. The high interest in these materials is explained by rich chemistry that enables tuning of their structural and optical characteristics. The holy grail property of these NPs is their fluorescence brightness, which can be 1000-fold higher than that of organic dyes, which opens new possibilities for biological and biomedical applications. In this tutorial review, we provide the first systematic analysis of brightness for a large variety of fluorescent organic nanomaterials. To this end, we discussed the definitions of brightness, which is essentially a product of extinction coefficient and fluorescence quantum yield of the nanoparticle. Moreover, we highlighted the importance to analyse brightness per volume, which allows comparison of brightness of NPs independently of their size. We described methods used for measuring and evaluating brightness of NPs in bulk (ensemble) and at the single-particle level. One should note that both types of measurements should be performed: while the first one provides a robust value directly linked to optical properties of NPs in solution, the latter shows the single-particle performance of NPs under the microscope. The discrepancy between these two methods frequently originates from >1000-fold higher excitation power (irradiance), used in the single-particle measurements. Then, we present current concepts to fight ACQ: aggregation-induced emission, the use of bulky side groups for neutral dyes and large hydrophobic counterions for ionic dyes. Moreover, the use of conjugated polymer NPs is a special approach, where the design combines conjugation of fluorophores through rigid pi-conjugated bonds and the use of bulky side groups.

The core of this review is dedicated to the presentation of the major classes of organic NPs and the systematic analysis of their properties. From this analysis it is clear that conjugated polymer NPs are among the brightest nanomaterials reported to date, however, they require a rather complex molecular design in combination with energy transfer. The future challenges here will be to further improve their extinction coefficient and develop a robust methodology to obtain small particle size, which is a key to achieve high fluorescence quantum yield. The AIE NPs showed relatively good brightness, however the current challenge in the field is to further increase the extinction coefficient of these dyes. A fruitful approach in this sense would be to combine flat, well-conjugated motifs with propeller-shaped AIE units. Neutral dyes with bulky side groups enable preparation of ultrabright macroscopic materials, but preparation of stable and bright NPs based on these fluorophores remains a challenge. Therefore, formulation of NPs and/or integration of these dyes into the polymeric matrix should be further improved. Finally, ionic dyes in combination with bulky hydrophobic counterions enabled preparation of ultrabright nanomaterials of different colour and size, having brightness per volume close to those of conjugated polymer NPs. This approach is particularly efficient because it uses cyanine and rhodamine derivatives, which are among the brightest dyes reported do date. However, bulky counterions present high molecular weight, which remains a limitation for further improvement of the per-volume brightness. Moreover, for all organic nanomaterials, the general challenge is to enhance their brightness in the NIR spectral regions, where all of them present relatively small fluorescence quantum yield. Overall, organic NPs show total brightness from 10^7^ till 10^9^ M^−1^ cm^−1^, which is ≥100 times higher than that of typical organic dyes. On the other hand, their per-volume brightness for the best examples ranges from 5000 to 12 000 M^−1^ cm^−1^ nm^−3^, which is still significantly lower compared to organic dyes in molecular form (*e.g. B*_V_ for rhodamine 6G is 10^5^ M^−1^ cm^−1^ nm^−3^). We expect that with proper molecular design and nano-formulation, these values can be further improved, reaching those reported for bulk materials. However, one should also note that the brightness of nanomaterials of a given size has a physical limit because of the Beer–Lambert law. Indeed, once the particle is capable to absorb close to 100% of photons, further enhancement of the extinction coefficient would be counterproductive. Moreover, in these cases the problem of re-absorption of emitted photons could occur, which would even decrease the overall brightness of the nanomaterial.

In bioimaging applications, brightness is particularly important for single-particle tracking, both at the cellular and small animal level. For intracellular applications, a compromise should be made with the particle size, which should be kept close to that of proteins. Brightness is also crucial for super-resolution imaging, where the number of photons improves both spatial and temporal resolution of the methods. The future developments here will focus on obtaining smallest possible NPs presenting highest possible brightness with or without blinking. Biosensing is a key application of fluorescent NPs, where the brightness affects directly the sensitivity of the method. Here, high brightness should be combined with proper functionalization of NPs, using for instance nucleic acids and proteins. Moreover, a variety of colours should be achieved for these NPs in order to access multiplexing capabilities. We expect that significant efforts will be dedicated in the coming years to bright fluorescent organic NPs as probes for biomolecular sensing based on FRET or affinity (binding) principles. Overall, it is clear that the future of organic nanomaterials is bright, but a lot more research is needed to make them indispensable tools in the biological and biomedical laboratories.

## Conflicts of interest

A. R. and A. S. K. are co-inventors of a filed patent applications on fluorescent polymeric nanoparticles and cofounders of BrightSens Diagnostics SAS. Other co-authors have no conflict of interest to disclose.

## Supplementary Material
